# Magnetotactic bacteria and magnetofossils: ecology, evolution and environmental implications

**DOI:** 10.1038/s41522-022-00304-0

**Published:** 2022-06-01

**Authors:** Pranami Goswami, Kuang He, Jinhua Li, Yongxin Pan, Andrew P. Roberts, Wei Lin

**Affiliations:** 1 Key Laboratory of Earth and Planetary Physics, Institute of Geology and Geophysics, Chinese Academy of Sciences, 100029 Beijing, China; 2 France-China Joint Laboratory for Evolution and Development of Magnetotactic Multicellular Organisms, Chinese Academy of Sciences, 100029 Beijing, China; 3 College of Earth and Planetary Sciences, University of Chinese Academy of Sciences, 100049 Beijing, China; 4 Research School of Earth Sciences, Australian National University, ACT, Canberra, ACT 2601 Australia; 5 Frontiers Science Center for Deep Ocean Multispheres and Earth System, Key Laboratory of Submarine Geosciences and Prospecting Techniques, MoE and College of Marine Geosciences, Ocean University of China, 266100 Qingdao, China

**Keywords:** Bacteria, Environmental microbiology

## Abstract

Magnetotactic bacteria (MTB) are a group of phylogenetically diverse and morphologically varied microorganisms with a magnetoresponsive capability called magnetotaxis or microbial magnetoreception. MTB are a distinctive constituent of the microbiome of aquatic ecosystems because they use Earth’s magnetic field to align themselves in a north or south facing direction and efficiently navigate to their favored microenvironments. They have been identified worldwide from diverse aquatic and waterlogged microbiomes, including freshwater, saline, brackish and marine ecosystems, and some extreme environments. MTB play important roles in the biogeochemical cycling of iron, sulphur, phosphorus, carbon and nitrogen in nature and have been recognized from in vitro cultures to sequester heavy metals like selenium, cadmium, and tellurium, which makes them prospective candidate organisms for aquatic pollution bioremediation. The role of MTB in environmental systems is not limited to their lifespan; after death, fossil magnetosomal magnetic nanoparticles (known as magnetofossils) are a promising proxy for recording paleoenvironmental change and geomagnetic field history. Here, we summarize the ecology, evolution, and environmental function of MTB and the paleoenvironmental implications of magnetofossils in light of recent discoveries.

## Introduction

**Fig. 1:**
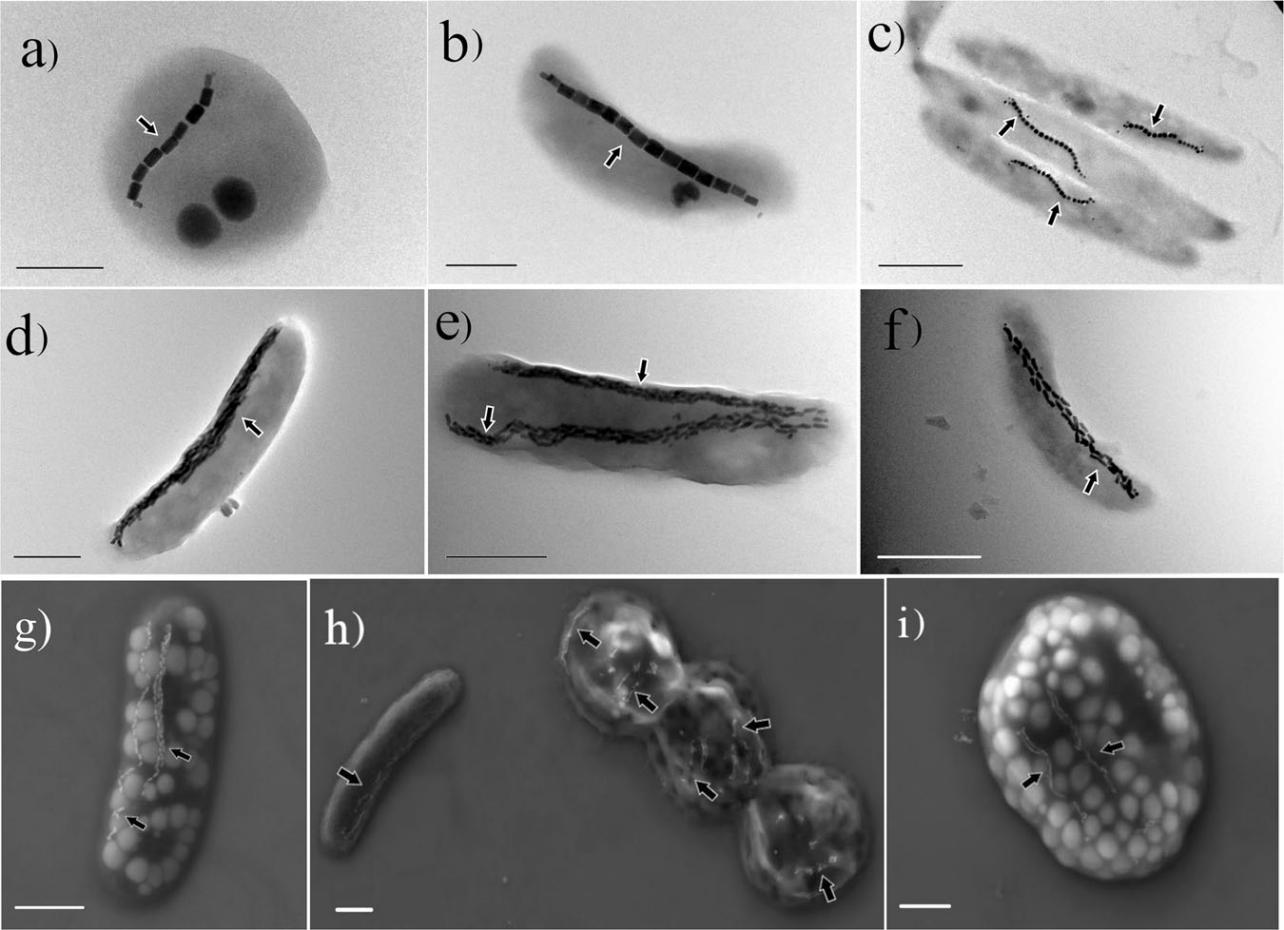
Electron microscope images of MTB. Transmission electron microscope (**a**–**f**) and scanning electron microscope (**g**–**i**) images of various MTB. Black arrows indicate magnetosome chains. Scale bars: **a**, **b** = 0.5 μm, **c**–**i** = 1 μm.

The terms biomineralization and magnetoreception are invariably associated with magnetotactic bacteria (MTB). Magnetoreception involves the use of Earth’s magnetic field for motility and navigation and biomineralization is a capability by which organisms produce minerals^1,2^. MTB are so far the only known group of prokaryotes with the ability to perform both biomineralization and magnetoreception^3^. MTB have been proposed to represent some of the most ancient organisms capable of biomineralization^4^. This group of bacteria was discovered in 1963 by Salvatore Bellini^5^, and was rediscovered independently in 1974 by Richard Blakemore^6^. The latter study also identified magnetosomal magnetic particles for the first time, which were referred to as “iron-rich particles”. MTB form magnetite (Fe_3_O_4_) and/or greigite (Fe_3_S_4_) crystals, generally in bead-like chains^7^ (Fig. 1). Magnetotaxis is one of their primary modes of motility^7^. Many organisms can use the geomagnetic field for navigation. Birds, fish, and certain migratory animals among others top this list, while desert ants, newts, spiny lobsters, snails, Bogong moths, and certain other invertebrates have been recently found to possess a magnetic sense of some sort^8–11^. Some mammals, too, can respond to Earth’s magnetic field. Laboratory experiments indicate that wood mice and mole rats make use of magnetic field lines while siting nests; certain cattle and deer use the field for body orientation while grazing; and dogs appear to orient toward north or south when they excrete^12–14^. Magnetic orientation is associated with many organisms, and understanding its origin is a subject of extensive research.

Magnetoreception represents a spectrum of capabilities of which magnetotaxis is a subset. While magnetoreception is mostly associated with higher organisms that use a magnetic sense for mobility, magnetotaxis is associated with microorganisms. It is related to previously discovered methods of taxis such as chemotaxis and aerotaxis, which are common modes of transportation and translocation by bacteria and archaea^15,16^. However, unlike chemotaxis or aerotaxis, which are multidirectional, magnetotaxis mostly involves upward/downward movement in search of optimal microenvironments near chemical gradients in water/sediment, aligning passively along Earth’s magnetic field^17^. It is thought that magnetotaxis along with chemotaxis/aerotaxis provides an additional benefit to MTB by permitting a one-dimensional search along the oxic–anoxic interface (OAI) in aquatic environments to enable MTB to find optimal oxygen concentrations to carry out necessary physiological functions^18^. MTB ecology, diversity, and evolution have been reviewed previously^19–21^; however, recent developments of omics, cultivation and magnetic measurements have expanded our understanding of MTB and magnetofossils. Multiple studies have pointed out that magnetotaxis is monophyletic in origin; that is, it originated from a single common ancestor^22–24^. This would make it a primordial physiological phenomenon and (probably) the earliest case of magnetoreception and systematic biomineralization on Earth^4,25^. Following this discovery, diverse multidisciplinary studies have sought to answer several significant questions. Are MTB widespread across the domain *Bacteria*? How did magnetotaxis originate and evolve? Do MTB have a significant role in biogeochemical element cycling? These questions are of multidisciplinary interest to microbiologists, geologists, physicists, and chemists. In this paper, we review progress to date in addressing these questions. We discuss the phylogenetic diversity of MTB and their potential role in the biogeochemical cycling of elements, and compare the metabolic pathways of all sixteen phylum-level MTB lineages identified so far. Moreover, we discuss expanding oxygen minimum zones (OMZs) in the oceans; diverse MTB likely live in OMZs and their environmental role in such settings has been understudied.

### MTB diversity and ecology

**Fig. 2:**
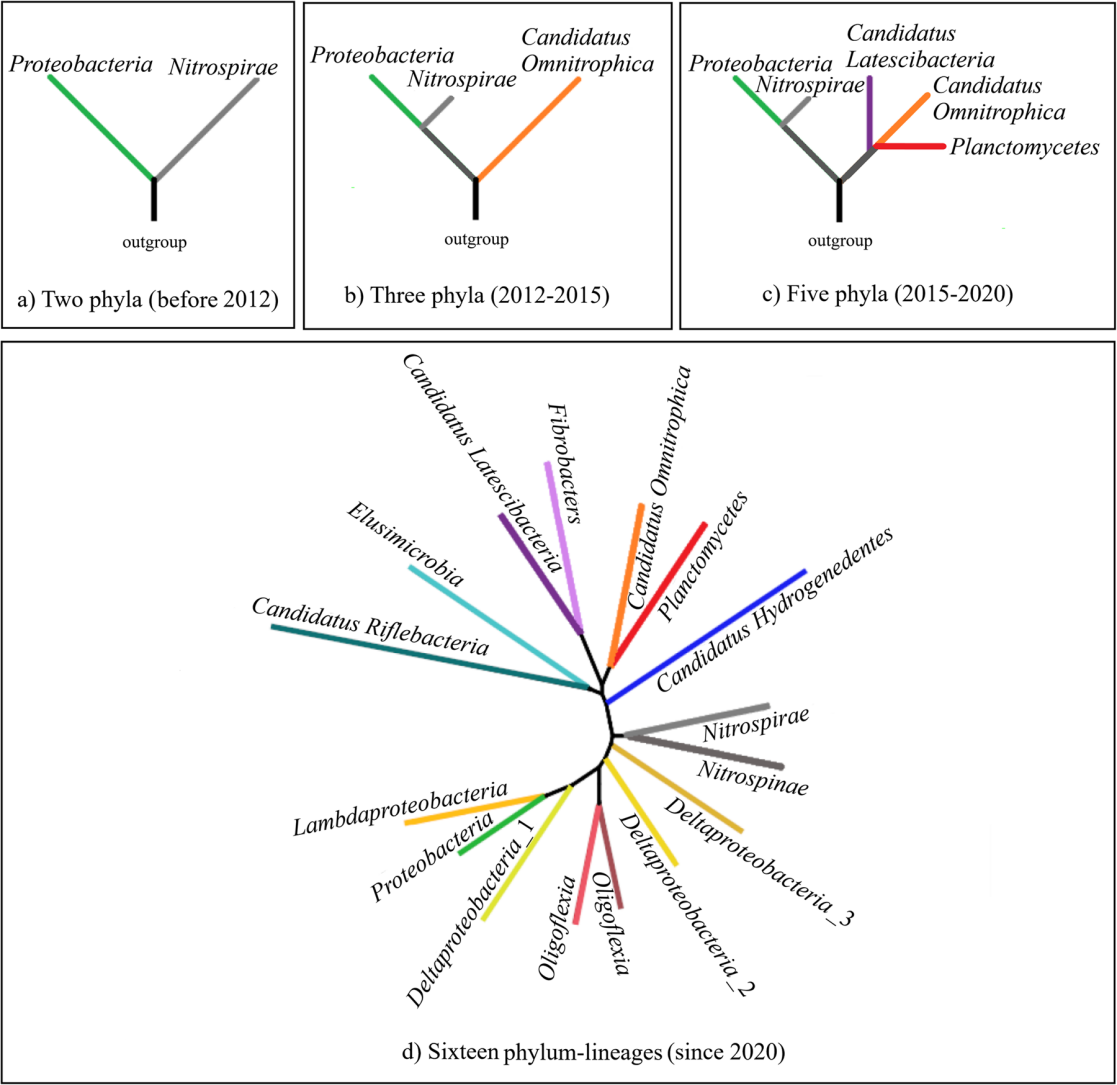
Schematic representation of the gradual expansion of the phylogenetic tree of MTB in the *Bacteria* domain. **a** Before 2012, only two phyla (*Proteobacteria* and *Nitrospirae*) were identified to contain MTB. **b** Between 2012 and 2015, an additional branch for the *Candidatus* Omnitrophica phylum was added^28^. **c** Subsequently, two bacterial phyla (*Candidatus* Latescibacteria^29^ and Planctomycetes^21^) were added to the MTB tree of life based on the presence of magnetosome gene clusters in their genomes. **d** The latest addition to the MTB tree has expanded its branches to a total of sixteen bacterial phylum-level lineages^30,31^. All taxonomic groupings are based on the NCBI taxonomy (https://www.ncbi.nlm.nih.gov/taxonomy).

MTB occur in multiple lineages of the bacterial tree of life, although with a patchy distribution. They include cocci, vibrio, rod, spirilla, and multicellular morphotypes^19,20,26,27^. Until 2012, both cultured and uncultured MTB could be grouped mostly into the *Proteobacteria* phylum (including the *Alphaproteobacteria*, *Gammaproteobacteria* and *Deltaproteobacteria* classes) and a few in the *Nitrospirae* phylum^27^ (Fig. 2a). In 2012, a novel uncultivated ovoid MTB (designated SKK-01) from Lake Chiemsee, Germany, was discovered and characterized to belong to the candidate phylum *Omnitrophica* (also known as candidate division OP3)^28^ (Fig. 2b). In 2015 and 2017, two additional bacterial phyla, the candidate phylum *Latescibacteria*^29^ and the phylum *Planctomycetes*^21^, respectively, were identified to contain MTB based on the presence of magnetosome gene clusters (MGCs, a group of genes responsible for magnetosome biomineralization and magnetotaxis) in their genomes (Fig. 2c). More recently, advances in metagenomic approaches, large-scale field studies and public database mining studies have expanded the total number of MTB-containing phylum-level lineages from five to sixteen^24,30,31^, which greatly increases the taxonomic and genomic diversity of MTB across the domain *Bacteria* (Fig. 2d). These findings highlight a much higher diversity of MTB lineages in the domain *Bacteria* than previously anticipated. On the basis of MGC types in MTB genomes, putative Fe_3_O_4_-producing MTB have been identified in all MTB lineages so far except for the *Latescibacteria* phylum, while putative Fe_3_S_4_-producing MTB exist in at least five bacterial phyla^31^.

MTB affiliated with the *Nitrospirae* phylum are biogeochemically important because many members of this group produce several hundred magnetosomal magnetite crystals per cell rather than the normal 10–50 crystals in most MTB^26,32^. Characteristic examples are the *Candidatus* Magnetobacterium bavaricum, which was discovered by Vali et al.^33^ and was subsequently phylogenetically defined by Spring et al.^26^ as magnetotactic rods that form elongated, bullet-shaped and kinked crystals arranged in a parallel chain-like fashion that appear as rope-like strands or bundles. Hanzlik et al.^34,35^ studied the chain architecture of *Ca*. Magnetobacterium bavaricum in detail. Apart from producing magnetite within magnetosomes, cells of *Ca*. Magnetobacterium bavaricum also harbor sulphur globules, solely comprising cyclo-octasulphur (S8), which gives them a probable role in both iron and sulphur cycling in nature^36^. The second example is *Ca*. Magnetobacterium casensis, which is smaller than *Ca*. Magnetobacterium bavaricum and produces around 200–500 magnetosomal Fe_3_O_4_ particles per cell^37–39^. Recent 16S rRNA-based and metagenomic surveys suggest that magnetotactic *Nitrospirae* are more diverse and more widely distributed than previously thought^31,40^, which emphasizes their environmental importance in various ecosystems.

Apart from single-celled bacteria, multicellular magnetic prokaryotes (MMPs) are flagella-aided motile organisms that also produce magnetic nanocrystals, as first reported by Farina et al.^41^. Despite their multicellular organization, MMPs have coherent magnetosome-chain polarity across their cells^41^. Many studies have now been carried out involving especially the spherical MMP morphotype (sMMP)^42–48^. A second morphotype, ellipsoidal MMP (eMMP), has been found in the Mediterranean Sea and Pacific Ocean^49–57^. MMPs are affiliated within the *Deltaproteobacteria* and MTB from this group produce Fe_3_O_4_ and/or Fe_3_S_4_ nanoparticles^23,31^. Studies of MMPs also provide insights into the origin and evolution of bacterial multicellularity, which is one of the most prevalent evolutionary innovations in the bacterial world^58^.

MTB have been identified from diverse biomes with highly variable, including extreme environments. For instance, MTB flourish in shallow hemipelagic sediments (at ~600 m depths) at 8 °C, in Santa Barbara Basin, Eastern Pacific Ocean^59^. Petermann and Bleil^60^ discovered live MTB from pelagic and hemipelagic sediments in the eastern South Atlantic Ocean. They observed assorted cocci, spirilla, vibrioids, and even rod-shaped MTB morphologies in pelagic environments on the African continental margin and on Walvis Ridge at ~3000 m and ~1000 m water depths, respectively. More recently, MTB have been observed from tropical marine environments in Singapore, which adds to equatorial MTB biodiversity^61^. A recent phylogenetic analysis of 16S rRNA gene sequences of microbial communities from a seamount in the Mariana volcanic arc (238–2023 m) near Challenger Deep, tropical western Pacific Ocean, revealed 16 novel MTB populations^62^. Abundant MTB have also been observed in seafloor sediments of the Yellow Sea with *Nitrospirae* being the dominant group^63^. In more extreme environments, Lefèvre et al.^64^ isolated three MTB strains of *Desulfonatronum thiodismutans* from hyper-alkaliphilic habitats in California, USA, that are obligate alkaliphiles (with optimal growth at pH 9.0–9.5) and sulphate reducers. Essential genes (i.e., dissimilatory sulphite reductase (*dsrAB*) and adenosine-5′-phosphate reductase (*aprA*)) were identified that verify that the novel strains are sulphate reducers. Psychrophilic MTB have been characterized from water and sediment samples from Admiralty Bay, King George Island, Antarctica, where average water temperatures during sampling ranged between 0.1 and 0.8 °C^65^. Nash et al.^66^ characterized a thermophilic MTB variety belonging to *Nitrospirae* from Little Hot Creek Hot Springs, California, and another *Gammaproteobacteria* MTB from the hyper-alkaline and hypersaline Mono Lake. Lefèvre et al.^67^ discovered a thermophilic MTB that they named *Candidatus* Thermomagnetovibrio paiutensis from mud and water samples in hot springs of the Great Boiling Springs geothermal field in Gerlach, northern Nevada. Two rod-shaped *Gammaproteobacteria* MTB strains, BW-2 and SS-5, from the hypersaline Salton Sea were the first isolated MTB from the *Gammaproteobacteria* class that biomineralize magnetite^68^. Further *Gammaproteobacteria* MTB strains FZSR-1 and FZSR-2 were identified recently from a salt evaporation pool in Fuzhou saltern, Bohai Bay, China^69^. Lin et al.^31^ also discovered living MTB cells in a relatively acidic peatland soil with draft genomes dominantly comprising magnetotactic *Nitrospirae*, *Omnitrophica* and *Deltaproteobacteria*.

### Origin and evolution of MTB

Ancient atmospheric molecular O_2_ in the Archean, 2.4 billion years ago (Ga), was less than 0.001% of the atmosphere today^70^ and Fe(II) concentrations probably ranged between 0.03 and 0.5 mM^71,72^ (modern oceanic iron concentrations range between 0.05 and 2.0 nM^73^). Archean ocean sulphate concentrations were also less than 1/100 of present levels, with concentrations lower than 200 µM^74,75^ and N_2_ was a bulk gas with similar levels to today^76^. Oxygen concentrations in the atmosphere and surface ocean first rose at ~2.4 Ga in the Great Oxygenation Event (GOE)^77^ with a second increase in the Neoproterozoic Oxygenation Event^78^ (NOE), which established a more modern ocean redox profile. The GOE is generally thought to have facilitated the emergence of eukaryotes^79^ while Betts et al.^80^ argue that the NOE was associated with emergence of large and complex multicellular organisms. Thus, the GOE and NOE were fundamental pacemakers for evolution of life on Earth, although the beginning of microbial life must have been much earlier than the GOE. There is evidence of microbial life originating sometime during the early Archean at ~3.5–3.8 Ga^79,81^. The presence of biogenic carbon has been uncovered in detrital zircon grains from Jack Hills, Western Australia^82^; if verified by further studies, the beginning of microbial life can be traced to ~4.1 Ga in the late Hadean Eon. Further evidence is required to corroborate this early age, which potentially transforms the way scientists view paleo-environments on Earth.

Fossilized magnetosomal magnetic particles (referred to as magnetofossils) can be preserved in sediments or rocks. Magnetofossil records trace an evolutionary history of MTB to the Cretaceous and with less certainty to the Paleoproterozoic at around ∼1.9 Ga^83–85^, which suggests that MTB should have an early origin. Furthermore, phylogenetic and molecular clock analyses suggest an Archean origin for genetic functionality of magnetosome biomineralization (which is needed to perform magnetotaxis); specifically, the emergence of MGCs is estimated to date to the Archean Eon (∼3.38–3.21 Ga)^25^ or even earlier^24,31^. It is important to remember that atmospheric oxygen was most likely sparse in the Archean so that an anoxic environment prevailed^86–89^. In addition to magnetotaxis, magnetosomal particles have been proposed to act as iron storage and sequestration organelles, or as gravity detection units or “geobatteries” that provided energy from elemental oxidation-reduction cycling^90^. It has recently been proposed that the initial role of magnetosomal magnetic particles was to mitigate intracellular reactive oxygen species (ROS) toxicity and that, eventually, they were co-opted for magnetotaxis in early Earth environments^4^. A photoferrotrophy-driven origin of magnetotaxis has also been proposed; that is, magnetosome formation was a by-product of Archean iron cycling and magnetotaxis evolved as a result of environmental pressure of co-evolved cyanobacteria and metabolically accumulated magnetite^91^. Magnetosomes have also been proposed to provide a protective shield in a metal-stressed environment^92^. To ascertain the evolutionary history of MTB, integration of microbiology, evolutionary biology, geobiology, biogeochemistry, and geochronology is required.

The recent development of metagenomics and single-cell genomics allows direct reconstruction of MTB genomes from environmental samples without cultivation. Comparison of these novel genomes with those from cultivated MTB strains provides insights into the evolution of magnetotaxis. Magnetosome protein phylogeny largely mirrors that of organisms at or above the class or phylum level, which suggests that vertical inheritance followed by multiple independent MGC losses mainly drove bacterial evolution of magnetotaxis at higher taxonomic levels^23,25,31^. Subsequent evolutionary trajectories of magnetotaxis at lower taxonomic ranks appear to be much more complicated and multiple evolutionary processes including horizontal gene transfers, gene duplications and/or gene losses may have been involved^93–95^. Metagenomic sequences with similarity to known magnetosome genes have been found in the microbiomes of some animals and even humans, which might suggest that MTB sensed by their hosts may produce symbiotic magnetoreception in these organisms^96–98^. It has also been suggested recently that the ability to biologically control magnetite precipitation might have been a fundamental feature of eukaryotic biology that was likely present in the last common ancestor of some archaea and extant eukaryotes^99^.

### Magnetic characterization and quantification of MTB and magnetofossils

Magnetofossils are distributed widely in freshwater and marine sediments^83^. Recently, their presence has also been reported in ferromanganese (Fe–Mn) crusts and abyssal manganese nodules^100–103^. Several methods exist for identifying magnetofossils. The most direct way is to observe them under a transmission electron microscope (TEM). Kirschvink and Chang^104^ first observed magnetofossils under a TEM from deep-sea sediments of the Southwest Atlantic, Equatorial Pacific, and South Atlantic Oceans. Petersen et al.^105^ found additional magnetofossil morphotypes from South Atlantic marine sediments, with bullet-, prismatic- and octahedral shapes that they considered to be the predominant natural remanent magnetization (NRM) carrier in sediments with ages from Quaternary to Eocene. Magnetosomal magnetite crystals usually have a [111] elongation direction, while Li et al.^106^ found that bullet-shaped biogenic magnetite grows in a two-stage process with the second stage involving elongation in the [100] direction. Kopp and Kirschvink^83^ proposed a series of criteria to identify magnetofossils. For instance, biogenic magnetosomal magnetite crystals have a narrow size distribution and distinctive morphologies with blunt crystal edges, high chemical purity, crystallographic perfection and chain arrangement.

Magnetic techniques provide complementary approaches that are commonly used to initially identify magnetofossils before direct TEM observation. These methods include low-temperature remanence measurements, first-order reversal curve (FORC) diagrams, and ferromagnetic resonance (FMR) spectroscopy. Moskowitz et al.^107^ suggested that low-temperature isothermal remanent magnetization (IRM) measurements can be used to distinguish magnetite magnetosome chains. They defined *δ* as *δ* = (IRM_80K_-IRM_150K_)/IRM_80K_ and the *δ* ratio = *δ*_FC_/*δ*_ZFC_, where *δ*_FC_ and *δ*_ZFC_ are the difference between field cooled (FC) and zero-field cooled (ZFC) IRM curves at 150 K and 80 K, respectively. Moskowitz et al.^107^ concluded that biogenic magnetite chains are present when the δ ratio >2. However, when magnetosome chains are oxidized, the *δ* ratio becomes less diagnostic. The test suggested by Chang et al.^108^, based on low-temperature cycling of a saturation IRM imparted at room temperature, is more robust for oxidized magnetosomes. Chang et al.^109^ reported that a double Verwey transition temperature signal can also be used to identify magnetofossils and suggested that ~100 K and ~120 K are the Verwey transition temperatures of biogenic and detrital magnetite, respectively, although it has also been suggested that the two Verwey transitions could result from other factors^110^.

FORC diagrams are normally interpreted in terms of the magnetic coercivity distribution and magnetostatic interactions among single-domain magnetic particles^111–113^. When interactions among uniaxial single-domain particles are negligible or weak, FORC diagrams will have a central ridge along the horizontal *B*_*c*_ axis, with a small vertical distribution along the vertical *B*_*u*_ axis. Although magnetic particles in magnetosome chains have strong interactions among them, the entire chain will act as a single needle with uniaxial magnetization that does not interact with other chains^114^. Therefore, MTB samples produce a central ridge in FORC diagrams^115–119^. However, when magnetofossil chains are broken, magnetic particles clump together so that interactions become strong; a central ridge usually persists and FORC distributions spread vertically in the *B*_*u*_ direction^120^. Inorganic magnetite with weak interactions also produce a central ridge FORC signature. Thus, if a FORC central ridge is observed, further TEM observations are needed to demonstrate the presence of magnetofossils.

**Fig. 3:**
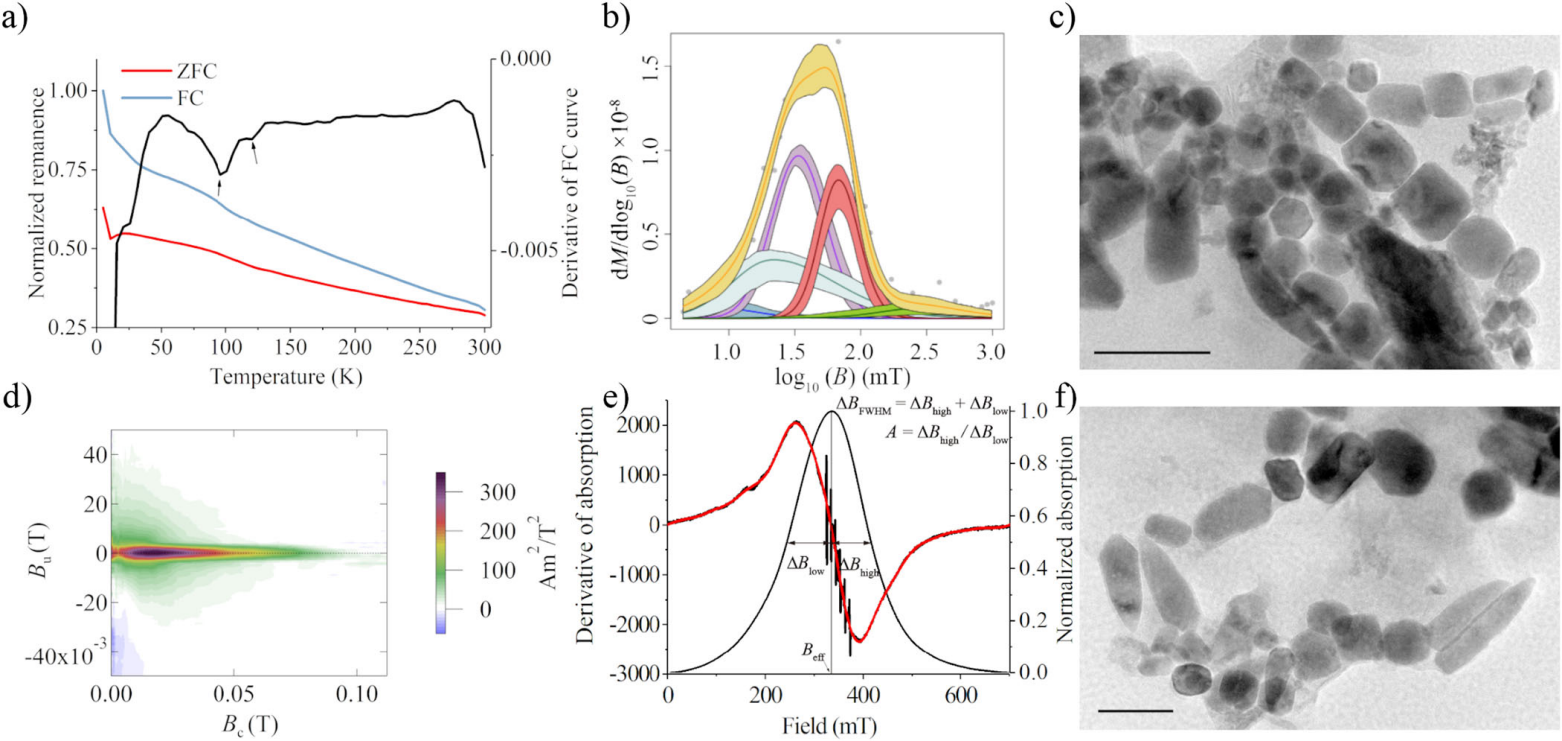
Methods for identifying magnetofossils in samples from core MD01-2444 at 26.74 m depth. **a** Low-temperature magnetic measurements (blue and red curves: normalized zero-field cooled (ZFC) and field cooled (FC) curves, respectively; black curve: normalized first derivative of the FC curve; peaks with arrows (~100 K and ~115 K) are indicative of biogenic and detrital magnetite, respectively). **b** Coercivity distribution from IRM acquisition curve unmixing (gray dots: IRM acquisition data; orange curve: spline fit based on measured data; purple and red curves: biogenic soft and biogenic hard components, respectively; shaded areas for each curve are 95% confidence intervals). **c**, **f** TEM images of magnetic extracts from the same sample. Scale bars: **c** = 200 nm, **f** = 100 nm. The main magnetofossil morphotypes are prisms, octahedra and bullets. **d** FORC diagram with a central ridge signature that is indicative of non-interacting single-domain magnetic particles. **e** Ferromagnetic resonance (FMR) and FMR absorption spectra (black curve: measured FMR spectrum; red curve: fitted FMR spectrum; black humped curve: normalized FMR absorption spectrum). Definition of commonly used magnetofossil-indicative parameters are shown in (**e**)^123^.

FMR spectroscopy is sensitive to the magnetic anisotropy of the chain configuration^121^. The magnetic anisotropy of MTB arises mainly from dipolar interactions among adjacent magnetic particles in a chain, which behaves like an elongated single-domain particle^114^; therefore, FMR is used to indicate the presence of magnetosome chains^122,123^. The main FMR parameters are as follows: the effective *g*-factor (*g*_eff_), asymmetry ratio (*A*), and empirical parameter (*α*), where *g*_eff_ = *hv*/*βB*_eff_, *h* is Planck’s constant, *v* is the microwave frequency, *β* is the Bohr magneton and *B*_eff_ is the maximum absorption field, *A* = Δ*B*_high_/Δ*B*_low_, where Δ*B*_high_ = *B*_high_ − *B*_eff_, and Δ*B*_low_ = *B*_eff_ − *B*_low_. The full width at half maximum is defined as Δ*B*_FWHM_ = *B*_high_ + *B*_low_ (Fig. 3e). Weiss et al.^122^ proposed that a magnetosome chain will have *A* < 1 and *g*_eff_ < 2.12. Kopp et al.^123^ proposed an empirical parameter, defined as *α* = 0.17 *A* + 9.8 × 10^−4^ Δ*B*_FWHM_/mT, and concluded that *A* < 1 and *g*_eff_ < 2.12 are insufficient to ensure the presence of magnetofossils. However, all of their measured MTB had *α* < 0.25, and magnetofossil-bearing samples have *α* = 0.25–0.30. When *α* is larger, magnetofossil contents are lower. Kodama et al.^124^ suggested that detrital and extracellularly produced authigenic magnetite (D + Ex) dominate sediments with *α* > 0.40, while magnetofossil-rich sediments have *α* < 0.35 and *A* < 1. However, inorganic elongated single-domain particles in a volcanic tuff also have 0.3 < *α* < 0.35 and *A* < 1^125^. Kind et al.^126^ investigated magnetic components in Holocene Lake sediments by combining anhysteretic remanent magnetization (ARM), IRM, FORC diagrams and FMR spectra and suggested a combination of FORC and FMR measurements to detect magnetofossils in natural environments. Blattmann et al.^127^ applied quantitative FMR to analyze magnetofossil variations in Lake Constance, which records sediment–water interface redox changes. FMR spectroscopy is also used widely to detect magnetofossils in pre-Quaternary marine sediments^116,128^.

To better understand the magnetic properties of magnetofossils, micromagnetic modeling has been applied to build a link between magnetofossil size distributions and magnetic properties. Muxworthy and Williams^129^ found that the largest reported interacting magnetosomal magnetite particles still have single-domain behavior based on micromagnetic simulations. Chang et al.^130^ used micromagnetic models to build a link between magnetic properties and magnetofossil size distributions from Ocean Drilling Program (ODP) Site 1263 during the Paleocene–Eocene thermal maximum (PETM). Moreover, micromagnetic modeling demonstrates that magnetosome-chain structures play a more important role in controlling magnetic particle coercivity than morphology^130^. Different MTB species have different chain structures and magnetic particle numbers. Therefore, magnetofossil diversity probably affects the magnetic properties of bulk samples, especially when magnetofossils are the main NRM carrier. Berndt et al.^131^ modeled magnetosome chain morphologies (including crystal size, elongation, chain length, and intra-chain spacing) and found that intra-chain spacing can control the magnetism of magnetosomal magnetic particles such as coercivity, coercivity of remanence, and saturation magnetization.

Heslop et al.^132^ calculated the vector difference sum (VDS) of components in NRM demagnetization curves to quantify magnetofossil contents from sediments offshore of northwestern Australia. In addition, IRM unmixing and principal component analyses applied to FORCs (FORC-PCA) are used to quantify magnetic components in sediments. Two categories of methods are used to unmix IRM acquisition curves. One is single sample IRM unmixing, which includes fitting of log-normal, skewed generalized Gaussian, and Burr-XII distribution models^133–135^. Egli^136^ concluded that the dispersion parameter of biogenic magnetite is <0.2, while values for detrital magnetite particles are between 0.3 and 0.4. Chang et al.^130^ analyzed magnetic components from PETM sediments using a cumulative log-Gaussian function and decomposed IRM acquisition curves into three components, including biogenic soft and hard magnetite, and a soft detrital component. They suggested that magnetofossil components contribute 76% of the total remanent magnetization during the PETM onset. A further IRM unmixing approach is endmember-based IRM unmixing, with non-negative matrix factoring^137^. Usui et al.^138^ reconstructed the biogenic proportion of particles using this approach and obtained a two biogenic magnetite endmember solution by combining χ_ARM_/IRM ratios (a magnetic mineral grain size proxy) and FORC diagrams. They concluded that the two biogenic magnetite endmembers represent isotropic and bullet-shaped magnetofossils.

Lascu et al.^139^ and Harrison et al.^140^ demonstrated that FORC-PCA can help to determine biogenic and detrital magnetite abundances. FORC diagrams enable the characterization of magnetic minerals with different domain states and interaction fields. Channell et al.^141^ applied this method to trace variations of three magnetic endmembers (EMs) in the Rockall Trough (NE Atlantic), including a magnetofossil EM. Roberts et al.^142^ used FORC-PCA to detect magnetic property variations during early diagenetic reduction, including loss of a magnetofossil component due to dissolution and to estimate the proportions of different minerals in different diagenetic systems. Yamazaki et al.^143^ applied FORC-PCA to two cores from the western North Pacific Ocean and the South Pacific Ocean and identified two non-interacting single-domain EMs, which represent biogenic soft and biogenic hard components, respectively^144^. Qian et al.^145^ used FORC-PCA analysis of eastern Mediterranean sediments to demonstrate that elevated magnetofossil abundances occur at oxidation fronts above organic-rich intervals. FORC-PCA is becoming increasingly common for quantifying sedimentary magnetofossil contents in sediments^146–148^. Combining magnetic measurements and TEM or scanning electron microscope (SEM) observations can provide information to identify, characterize and quantify magnetofossils in natural samples (Fig. 3).

### Paleoenvironmental and paleomagnetic implications of magnetofossils

Varying proportions of different magnetofossil morphotypes can reflect the paleoredox level of sediments^100,101,103,149–151^. Abundant bullet-shaped magnetofossils have been detected in reducing environments with higher total organic carbon (TOC), while octahedral magnetofossils appear to dominate relatively oxic environments. However, Lean and McCave^152^ found that more elongated magnetofossils occur in low-TOC horizons. Therefore, the paleoenvironmental implications of magnetofossil morphologies need further investigation, especially the relationship between magnetofossil abundance and sediment nutrient content. Moreover, if magnetofossils undergo diagenetic modification, the relationship between their abundance and paleoenvironment will change^153^. For example, moderate TOC availability promotes mild diagenetic iron reduction that reduces sedimentary iron so that it becomes bioavailable to MTB^116^. However, high TOC produces sulphidic diagenetic environments in which magnetite magnetofossils dissolve^154^. Equant magnetofossils dissolve more easily than bullet-shaped forms^155^, and bullet-shaped magnetofossils are more easily dissolved than hexagonal prisms and octahedral forms^153^. Magnetofossils are ideal ancient magnetic field recorders. In environments where magnetofossils are preserved, Ouyang et al.^156^ and Chen et al.^157^ demonstrated that that they have a higher magnetic recording efficiency than detrital magnetite, while Li et al.^158^ found that biogenic magnetite can be less efficiently magnetized than detrital magnetite. The debate also remains about whether magnetofossils record a biogeochemical remanent magnetization (BGRM). Tarduno et al.^159^ suggested that magnetofossils that survive burial below the Fe-redox boundary can produce delayed BGRM acquisition. Roberts et al.^116^ noted that paleomagnetic signals carried by magnetofossils must be acquired at shallow depths based on comparison between two nearby records. Tests for offsets between paleomagnetic signals carried by detrital and biogenic magnetite indicate no depth lag, so the evidence is still lacking for the BGRM mechanism^156,157^. Yamazaki et al.^160^ found that elongated magnetofossils inhabit lower parts of a redox zonation, while isotropic forms occupy shallower levels.

Although there are few reports of greigite magnetofossils, it has been suggested that they can be reliable paleomagnetic recorders^161^. Pósfai et al.^162^ proposed that greigite in Miocene sedimentary rocks is similar to biogenic crystals produced by MMP and Chang et al.^163^ concluded that greigite magnetofossils are potentially widespread in ancient sediments. The favored MTB habitat depth relates to their ability to contribute to a BGRM; future detailed studies of the vertical distribution of MTB populations within the water column or sediments are required to constrain the habitat range of MTB.

### Ecosystem functions of MTB

**Fig. 4:**
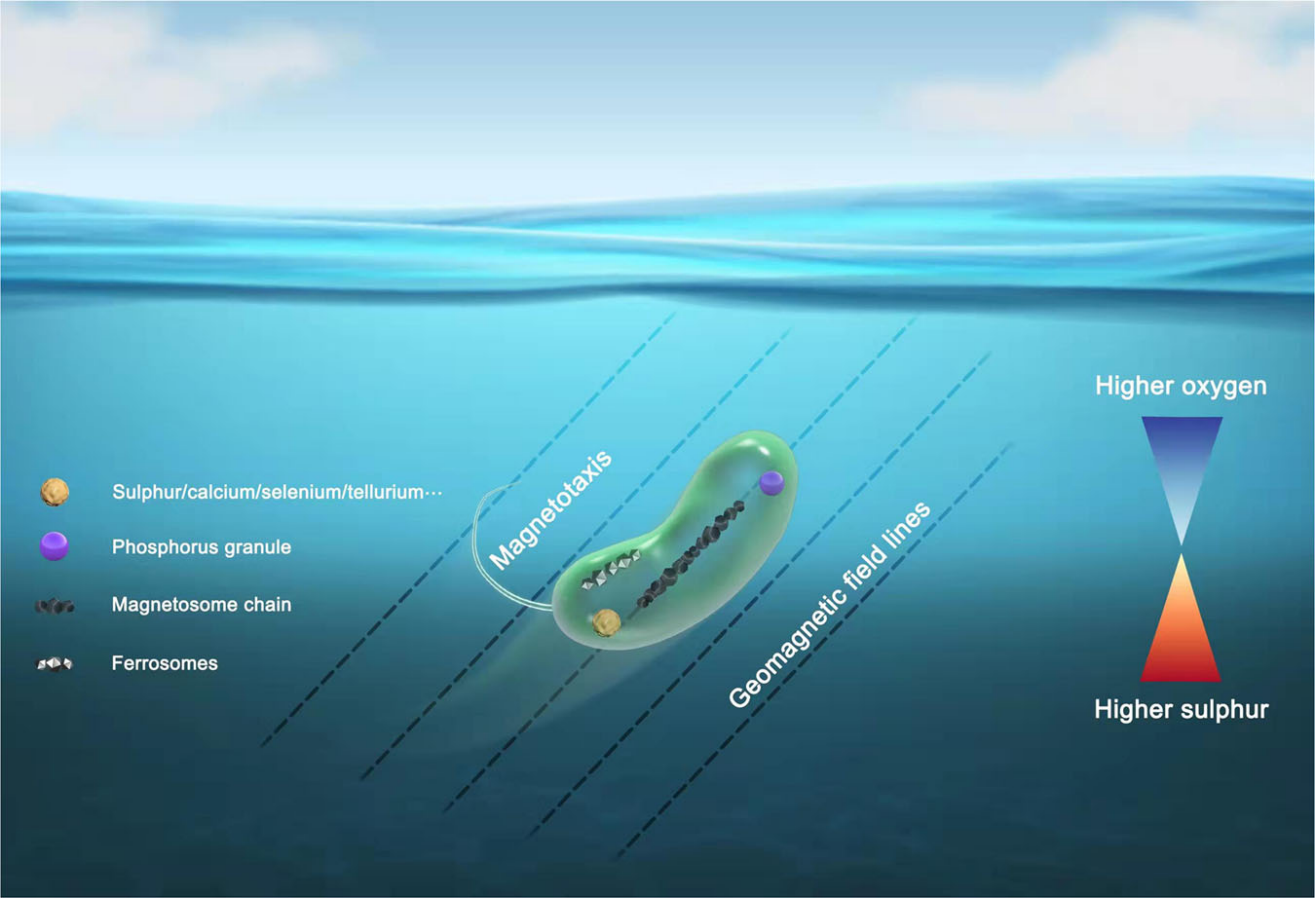
Magnetotaxis, combined with chemotaxis and aerotaxis, allows MTB to efficiently locate and maintain an optimal position for survival and growth in habitats with vertical redox concentration gradients in water columns and sediments around an oxic–anoxic interface (OAI). MTB are widely distributed in aquatic environments from marine to freshwater ecosystems and are thought to play important roles in the cycling of various elements (e.g., Fe, C, N, S, P, and some heavy metals, such as tellurium and selenium).

**Fig. 5:**
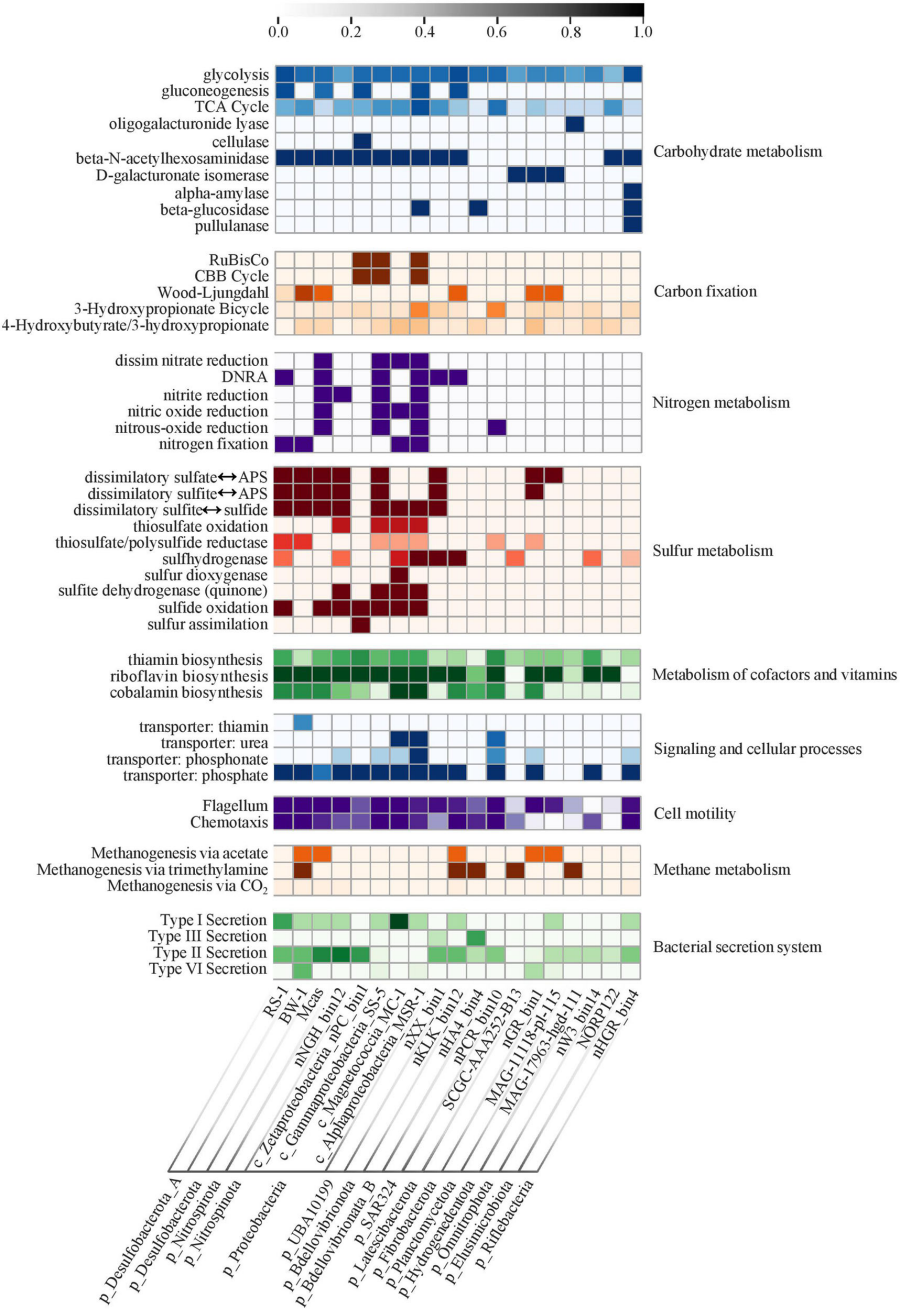
Metabolic pathways of representative MTB genomes across 16 bacterial phylum-level lineages. The color gradient represents the completeness of major metabolic pathways inferred from the presence or absence of genes. Dark represents a complete or nearly complete pathway, and white represents a pathway that is absent or mainly incomplete. Note that most available MTB genomes are draft or incomplete, so we cannot rule out that the absence or incompleteness of metabolic pathways maybe due to the fragmented nature of draft genomes. All taxonomic groupings are based on the GTDB taxonomy (Release 89, https://gtdb.ecogenomic.org).

MTB are distributed globally in aquatic ecosystems^21^. They can comprise up to ~30% of the microbiome in some habitats^26^ and their biomineralizing capability is assumed to be important for biogeochemical cycling of iron in sediments^90^. The recent discovery of an Archean origin for MTB further suggests that they may have contributed to iron cycling throughout Earth history. With reports of the ability of MTB to fix nitrogen^164,165^, sequester carbon^166^, and to incorporate elements like sulphur, phosphorus, selenium, calcium, etc., into biominerals (Fig. 4)^28,32,39,57,64,95,166–171^, evidence is growing to indicate that MTB play important roles in global biogeochemical cycling. Here, we compared the major KEGG pathways of representative MTB genomes across all known 16 bacterial phylum-level lineages, which suggest that MTB have phylogenetically diverse metabolic features (Fig. 5). What is not yet clear is the extent to which they impact the total cycling budget of each element.

To better understand iron biomineralization, the processes and products of biomineralization, the relevant genetics, and magnetotaxis need to be analyzed. The ability to navigate by magnetotaxis depends on whether magnetosomal magnetite particles are fully developed^19^. Magnetotaxis is best achieved with fully developed crystals (~30–150 nm)^7,59,172^ rather than with immature crystals (~30 nm or smaller). Cornejo et al.^172^ studied the biomineralization mechanism for mature octahedral crystal formation inside magnetosome membranes in *Magnetospirillum magneticum* strain AMB-1 (AMB-1), whereas Li et al.^173^ studied the mechanism for mature bullet-shaped crystal formation in *Deltaproteobacterial* strain WYHR-1. These and further studies could help to better understand crystal biomineralization in various MTB strains. Magnetic particle growth is important because assemblages of mature magnetosomal magnetite particles in chain-like structures produce an intracellular magnetic dipole that interacts with Earth’s magnetic field to assist MTB cells in magnetic navigation^114,174–176^. Therefore, research on the growth stage of magnetosomal crystals in different MTB populations would help to better understand underlying magnetotaxis mechanisms.

Bioavailable iron is scarce in many environments today, which may limit MTB population growth^113^. In particular, iron is scarce in high-nitrate low-chlorophyll (HNLC) oceans. Addition of iron from, for example, eolian dust or volcanic ash inputs, can fertilize surface ocean primary productivity, which increases the chlorophyll content in iron-limited biomes^177–179^. Solubilization of iron from such inputs plays a key role in carbon and nitrogen fixation into biomass, which makes iron bioavailability responsible for primary production in these ecosystems^180–183^. Export of carbon from the surface ocean to the seafloor can also stimulate mild diagenetic release of iron in the uppermost sediment to release a limitation on MTB productivity in pelagic environments and allows MTB populations to grow^116^. Considering the global MTB distribution in aquatic ecosystems, it has been suggested that MTB communities play significant roles in present-day global iron cycling^20,184^.

Apart from MTB, magnetic inclusions have been reported within the purple-sulphur bacterial genera, *Rhodopseudomonas* and *Ectothiorhodospira*^185^. These inclusions have no prismatic crystal structure and they lack a lipid-membrane to enclose magnetosomes. Unlike the microaerophilic model strains, AMB-1 and *Magnetospirillum gryphiswaldense* strain MSR-1 (MSR-1), recent analysis of an anaerobic sulphate-reducer, *Desulfuvibrio magneticus* strain RS-1 (RS-1), is gaining popularity as a model MTB organism^186,187^. This species constructs sub-cellular electron-dense Fe-rich granules (sometimes including phosphorus and oxygen) that are encased by a lipid-like membrane. These “ferrosome” (iron body) granules are independent and separate entities from magnetosomes and are proposed to play a vital role in storing iron under anaerobic respiration during extreme iron starvation conditions^188^. This aligns with the findings of Amor et al. that magnetite in AMB-1 only represents about ~25–30% of the total iron mass in a bacterial cell^189^. Broader iron storage remains to be explored in relation to other MTB species to explore their probably crucial role in global iron cycling.

Apart from the well-studied Fe_3_O_4_ and Fe_3_S_4_ biomineralization products within MTB, diverse other inclusions contain elemental sulphur, polyphosphate, calcium, etc. Sulphur-rich inclusions have been identified in *Magnetovibrio* strains MV-1 and MV-2 of the *Alphaproteobacteria* class and in many *Nitrospirae* MTB^7,28,32,57,190,191^. It was initially proposed that some MTB, like *Nitrospirae* MTB, are capable of adapting to chemical gradients near the OAI by adjusting their metabolic strategies—either by moving downward to accumulate reduced sulphur species or upward to oxidize stored sulphur with oxygen^192^. This redox-controlled response is supported by analysis of the vertical distribution of *Ca*. Magnetobacterium bavaricum in sediments^32^ and by genomic characterization of *Ca*. Magnetobacterium casensis^38^. *Ca*. Magnetobacterium casensis and *Ca*. Magnetobacterium bavaricum are proposed to conduct sulphur oxidation with nitrate and/or oxygen as electron acceptors in the upper micro-oxic layer and to perform sulphate reduction in the anoxic lower layer, suggesting a complex metabolic strategy of *Nitrospirae* MTB and electron shuttling depending on redox conditions^38,39,193^.

Bazylinski and Blakemore^164^ revealed that *Magnetospirillum magnetotacticum* strain MS-1 (MS-1) fixes nitrogen at rates equivalent to those of *Azospirillum lipoferum* when cultured microaerobically under nitrogen limiting conditions, where *Azospirillum* species were among the earliest known potential nitrogen fixers for cereal plants^194^. Apart from MS-1, two *Magnetospirillum* strains, including MSR-1 and AMB-1, when proliferated in nitrogen limiting, semi-solid media can also fix nitrogen via nitrogenase^164^. MSR-1 contains indispensable nitrogenase structural genes required for nitrogen fixation in the presence of the DRAT-DRAG nitrogenase post-translational regulatory system^165^ (Fig. 5).

Carbon sequestration is a key consideration when analyzing pollution mitigation potency of a plant or microorganism, both in terrestrial and aquatic habitats. Microorganisms and other autotrophs drive the carbon cycle because they are at the forefront of carbon cycle feedback mechanisms and their primary production is critical in carbon cycling^195^. Marine phytoplankton have a well-known role in CO_2_ sequestration^196^; chemolithoautotrophs do the same under dark conditions in deeper, less-oxic/anoxic parts of ocean water or sediment redox gradients^197^. If not obligate, most MTB are facultative chemolithoautotrophs, which might suggest an underlying role in CO_2_ fixation^19^. Most terrestrial plants and microorganisms contain genes that encode a key metabolic pathway known as the Calvin–Benson–Bassham pathway (CBB) that allows the plant/microbe to sequester bioavailable carbon from the environment. Ribulose-1,5-bisphosphate carboxylase/oxygenase (RuBisCO) is an abundant protein that catalyzes the CO_2_ fixation reaction^198^. The *cbbM* gene that encodes form II RuBisCO was identified in magnetotactic chemolithoautotrophs *Magnetovibrio* strains MV-1 and MV-2 and *Magnetospirillum* strain MS-1; these species are proposed to be important in carbon cycling and primary productivity^166^. *Gammaproteobacteria* MTB strains BW-2 and SS-5 from the hypersaline Salton Sea have a partial RuBisCO gene sequence and potentially use the CBB pathway for autotrophy^68^. MTB from the *Etaproteobacteria* (*Magnetococcus marinus* strain MC-1) use the reverse tricarboxylic acid (rTCA) cycle for autotrophy^199^, while magnetotactic *Nitrospirae* are proposed to fix CO_2_ via the reductive acetyl-CoA (Wood-Ljungdahl or WL) and/or reductive tricarboxylic acid (rTCA) pathways^38,193,200^ (Fig. 5).

Uncultured MTB cells from the Seine River, France, contain vesicles rich in barium and calcium oxide^171^ and MTB from Lake Pavin, France, biomineralize CaCO_3_ in addition to magnetosomal magnetite^201^. Instances of phosphorous sequestration by *Magnetococcaceae* alongside magnetite chains have also been demonstrated in ferruginous Lake Pavin^168^ and in the anoxic Black Sea^169^. Schulz-Vogt et al.^169^ suggested that magnetotactic cocci act as a shuttle (using magnetotaxis) to transport phosphorous from the upper to the lower stratum of the suboxic zone, which prevents eutrophication resulting from excess phosphorus accumulation in the upper stratum.

Elements can also deposit on MTB cell surfaces, which is important in analyzing the role of MTB in heavy-metal bioremediation and magnetic separation of metal-loaded MTB cells from aquatic bodies. For example, Arakaki and colleagues^202^ observed electron-dense Cd^2+^ deposits enveloping RS-1 cell surfaces under TEM when cells were cultured in growth media containing 1.3 ppm Cd^2+^. Mono-dispersed crystalline inclusions have 20–40 nm sizes and were easily distinguished from magnetic particles in TEM images^202^. A novel *Alphaproteobacterium* MTB species grown in a cobalt supplemented medium has efficient biosorption competence with 89% cobalt removed by magnetic separation of the biomass^203^. Tellurite (O_3_Te^2−^) uptake by MTB followed by biomineralization of discrete tellurium crystals alongside and separate from magnetite crystals was observed by Tanaka et al.^204^. These authors then demonstrated that when a growth medium is supplemented with selenite (SeO_3_^2−^), elemental selenium nanoparticles with no definitive form gradually built up and some precipitated within cells, in conjunction with and autonomous from magnetite particles^170^. Quantitative analysis revealed that MTB accumulate selenium at a higher rate per cell than other non-iron element accumulations. For example, Se accumulated by MTB is higher than O_3_Te^2−^ uptake and Cd adsorbed by factors of 2.4 and 174, respectively^170^. Elemental adsorption on cell surfaces is not specific to MTB, but they can contribute to metal pollutant removal via magnetic separation. Heavy-metal recovery from diodes and resistors of waste printed circuit boards using MTB is a latest discovery in terms of MTB applications in bioremediation^205^. Although such high metal concentrations are usually not encountered in natural environments, analysis of samples from polluted water bodies will help to identify the usefulness of MTB for bioremediation, e.g., MTB morphotypes have been observed in hydrocarbon-contaminated microcosms from the Gulf of Fos, France, and hypothesized to be involved in degrading hydrocarbons or other aromatic compounds^206^. It remains to be shown if MTB extracted from such microcosms can be grown in defined induction experiments using aromatic substrates under anaerobic conditions. MTB could, therefore, be potent model organisms for bioremediating contaminated water bodies and surface sediment. As new MTB genomes are obtained by metagenomics and single-cell genomics of environmental samples, identification and characterization of key proteins involved in these metabolic pathways will provide an important way to understand the ecological functions of MTB.

### MTB as a potential important constituent of OMZ microbiomes

Oxygen minimum zones (OMZs) are oxygen delimiting/nutrient-rich sections of an oceanic gradient^207^. Waters above and below the OMZ have higher oxygen concentrations compared to the OMZ interval. OMZs are global sinks for reactive nitrogen because they have the highest microbial activity that conserves available oxygen and produces N_2_ and N_2_O as respiration by-products^208^ and have been termed “microbial reactors” with global significance^209^. OMZs have expanded gradually over the past 50 years due to ocean warming, which reduces oxygen solubility^210–212^. Microbiological analyses of oceanic OMZs reveal that the most abundant phyla are *Proteobacteria*, *Bacteroidetes*, *Actinobacteria*, *Planctomycetes,* and *Marinimicrobia* (previously known as Marine Group A)^213^. A characteristic feature of the OMZ microbiome is its predominant role in the biogeochemical cycling of marine nitrogen^214,215^, sulphur^216–219^, and carbon^215^. Rhoads et al.^220^ showed that MTB can occur within an OMZ at oxygen levels as low as <4 mg L^−1^. The range of dissolved oxygen contents in an OMZ appears to be optimal for MTB to thrive (0.1–0.5 mg L^−1^). It has been hypothesized that marine dysaerobic zones (with 0.1–0.5 mL L^−1^ dissolved oxygen concentrations) have a biogenic magnetite inventory based on TEM results in which fine-grained (80–100 nm) subrounded cubic magnetite predominates^220^. High-nitrate concentrations in OMZs suggest that these oxygen-limiting microenvironments should harbor greater denitrifying MTB populations^220^. Symbiotic occurrences have been proven by studying the “cryptic” sulphur cycle in OMZs around the world^221^. OMZ biogeochemistry is important in the greater oceanic system with implications even outside the oceans. For example, consortia of ectosymbiotic, sulfate-reducing, chemolithoautotrophic *Deltaproteobacteria*, and Excavata protists have been reported^222^. Sulphur-reducing bacteria with magnetosomes have also been observed in microbial mats on carbonate concretions from the Black Sea alongside other archaea that aid methane oxidation^223^. Sulphur-reducing gammaproteobacterial MTB even occur as extracellular symbionts on marine bivalves^224^. OAIs and OMZs are ecological niches in which MTB thrive within chemical gradients; tweaking the chemistry of such environments will likely affect much of the microbial community. The increasing importance of OMZs in the modern ocean could imply that global MTB populations will increase over time with accelerating ocean warming and that they could help to limit deterioration of ocean ecosystem health.

### Outlook

MTB are distributed in aquatic ecosystems globally. Understanding of the phylogenetic taxonomy and metabolic flexibility of MTB has expanded greatly over the past decade, which suggests an unexpected natural diversity of these microorganisms. Continued discovery of novel MTB lineages from different environments highlights our limited knowledge of their diversity and ecology. Continuing cultivation-dependent and -independent MTB analyses will be crucial to understand their diversity and taxonomy more fully. We anticipate great progress in defining the phylogenetic diversity and evolutionary origin of MTB in the coming years. MTB incorporate elements from their surroundings to produce several biomineral types, with magnetic iron-bearing minerals being the primary biomineralization product. In addition to iron biomineralization, MTB can fix nitrogen, oxidize/reduce sulphur and sequester carbon and phosphorus from the environment. These findings suggest that MTB play important roles in biogeochemical elemental cycling through time, although their contributions are yet to be evaluated quantitatively. Moreover, MTB in marine OMZs/OAIs may play a role in ocean biogeochemical cycling and, in turn, in trimming eutrophication. If this is demonstrated by further work, MTB could be a natural eutrophication repressor that could become important with ongoing global warming. In addition, MTB can be used in pollution bioremediation by accumulating heavy metals on their surfaces by adsorption (e.g., Cd) and intracellularly (e.g., O_3_Te^2−^ and Se). The magnetism of MTB ensures that metal-loaded MTB cells can be separated magnetically from contaminated waters. Magnetofossils are important in paleoenvironmental, paleoclimatic and paleomagnetic interpretations of sediments and sedimentary rocks. Further research is needed to understand the environmental adaptability of MTB and limiting factors that affect magnetofossil preservation to develop robust magnetofossil proxies for past environmental changes.

## Data Availability

All NCBI accession numbers of MTB genomes used in Fig. 5 are as follows-HG794546.1: Magnetospirillum gryphiswaldense MSR-1; CP000471.1: Magnetococcus marinus MC-1; CP032508.2: Gamma proteobacterium SS-5; JADFZC010000123.1: Oligoflexales bacterium isolate nHA4_bin4; JADFZW010000010.1: Oligoflexia bacterium isolate nKLK_bin12; AP010904.1: Desulfovibrio magneticus RS-1; FWEV01000001.1: Candidatus Desulfamplus magnetomortis BW-1 PRJEB14757; NVTF01000001.1: Elusimicrobia bacterium isolate NORP122; JADFYW010000100.1: Fibrobacteria bacterium isolate nGR_bin1; DUZM01000001.1: Candidatus Hydrogenedentes bacterium isolate MAG_17963_hgd_111; ASWY01000001.1: Latescibacteria bacterium SCGC AAA252-B13; JADGAO010000100.1: Nitrospinae bacterium isolate nNGH_bin12; JMFO00000000: Candidatus Magnetobacterium casensis; JADGBR010000010.1: Candidatus Omnitrophica bacterium isolate nW3_bin14; DUZJ01000001.1: Planctomycetes bacterium isolate MAG_11118_pl_115; JADGAS010000010.1: Zetaproteobacteria bacterium isolate nPC_bin1; JADFZL010000010.1: Candidatus Riflebacteria bacterium isolate nHGR_bin4; JADGAT010000100.1: SAR324 cluster bacterium isolate nPCR_bin10; JADGCS010000100.1: Deltaproteobacteria bacterium isolate nXX_bin1. The original data for Fig. 3 can be found at 10.6084/m9.figshare.17122277.v1.

## Code availability

MagCluster (https://github.com/RunJiaJi/magcluster), code: magcluster prokka 19mtb_genomes; KofamKOALA server (https://www.genome.jp/tools/kofamkoala/) 10.1093/bioinformatics/btz859KEGG-Decoder10.1038/s41396-018-0091-3, code: KEGG-decoder -input 19mtb.txt -output out.list -vizoption static -m myorder.txt. Fig. 3b was produced using by Max UnMix and Fig. 3d was drawn using FORCinel v3.06 (https://wserv4.esc.cam.ac.uk/nanopaleomag/?page_id=51).

## References

[1] Kirschvink, J. L., Jones, D. S. & MacFadden, B. J. *Magnetite biomineralization and magnetoreception in organisms* (Springer US, 1985).

[2] Frankel, R. B. & Blakemore, R. P. *Iron Biominerals* (Springer US, 1991).

[3] Uebe R, Schüler D (2016). Magnetosome biogenesis in magnetotactic bacteria. Nat. Rev. Microbiol..

[4] Lin W, Kirschvink JL, Paterson GA, Bazylinski DA, Pan Y (2020). On the origin of microbial magnetoreception. Natl Sci. Rev..

[5] Bellini S (2009). On a unique behavior of freshwater bacteria. Chin. J. Oceanol. Limnol..

[6] Blakemore RP (1975). Magnetotactic bacteria. Science.

[7] Bazylinski DA, Frankel RB (2004). Magnetosome formation in prokaryotes. Nat. Rev. Microbiol..

[8] Boles LC, Lohmann KJ (2003). True navigation and magnetic maps in spiny lobsters. Nature.

[9] Nordmann GC, Hochstoeger T, Keays DA (2017). Unsolved mysteries: Magnetoreception—a sense without a receptor. PLoS Biol..

[10] Wajnberg E (2010). Magnetoreception in eusocial insects: an update. J. R. Soc. Interface.

[11] Dreyer D (2018). The Earth’s magnetic field and visual landmarks steer migratory flight behavior in the nocturnal Australian Bogong moth. Curr. Biol..

[12] Malkemper EP (2015). Magnetoreception in the wood mouse (*Apodemus sylvaticus*): influence of weak frequency-modulated radio frequency fields. Sci. Rep..

[13] Begall S, Malkemper EP, Cervený J, Němec P, Burda H (2013). Magnetic alignment in mammals and other animals. Mamm. Biol..

[14] Hart V (2013). Dogs are sensitive to small variations of the Earth’s magnetic field. Front. Zool..

[15] Engelmann TW (1881). Neue methode zur untersuchung der sauerstoffausscheidung pflanzlicher und thierischer organismen.. Pflüger Arch. Gesammt. Physiol. Menschen Thiere.

[16] Adler J (1966). Chemotaxis in bacteria. Science.

[17] Vali, H. & Kirschvink, J. L. Observations of magnetosome organization, surface structure, and iron biomineralization of undescribed magnetic bacteria: evolutionary speculations. In *Iron Biominerals* (eds. Frankel, R. B. & Blakemore, R. P.) 97–115 (Springer US, 1991).

[18] Mao X, Egli R, Petersen N, Hanzlik M, Liu X (2014). Magneto-chemotaxis in sediment: first insights. PLoS ONE.

[19] Lefèvre CT, Bazylinski DA (2013). Ecology, diversity, and evolution of magnetotactic bacteria. Microbiol. Mol. Biol. Rev..

[20] Lin W, Bazylinski DA, Xiao T, Wu LF, Pan Y (2014). Life with compass: diversity and biogeography of magnetotactic bacteria. Environ. Microbiol..

[21] Lin W, Pan Y, Bazylinski DA (2017). Diversity and ecology of and biomineralization by magnetotactic bacteria. Environ. Microbiol. Rep..

[22] Abreu F (2011). Common ancestry of iron oxide- and iron-sulfide-based biomineralization in magnetotactic bacteria. ISME J..

[23] Lefèvre CT (2013). Monophyletic origin of magnetotaxis and the first magnetosomes. Environ. Microbiol..

[24] Lin W (2018). Genomic expansion of magnetotactic bacteria reveals an early common origin of magnetotaxis with lineage-specific evolution. ISME J..

[25] Lin W (2017). Origin of microbial biomineralization and magnetotaxis during the Archean. Proc. Natl Acad. Sci. USA.

[26] Spring S (1993). Dominating role of an unusual magnetotactic bacterium in the microaerobic zone of a freshwater sediment. Appl. Environ. Microbiol..

[27] Amann, R., Peplies, J. & Schüler, D. Diversity and taxonomy of magnetotactic bacteria. In *Magnetoreception and Magnetosomes in Bacteria* (ed. Schüler, D.) 25–36 (Springer, 2006).

[28] Kolinko S (2012). Single-cell analysis reveals a novel uncultivated magnetotactic bacterium within the candidate division OP3. Environ. Microbiol..

[29] Lin W, Pan Y (2015). A putative greigite-type magnetosome gene cluster from the candidate phylum. Latescibacteria. Environ. Microbiol. Rep..

[30] Uzun M, Alekseeva L, Krutkina M, Koziaeva V, Grouzdev D (2020). Unravelling the diversity of magnetotactic bacteria through analysis of open genomic databases. Sci. Data.

[31] Lin W (2020). Expanding magnetic organelle biogenesis in the domain Bacteria. Microbiome.

[32] Jogler C (2010). Cultivation-independent characterization of ‘*Candidatus* Magnetobacterium bavaricum’ via ultrastructural, geochemical, ecological and metagenomic methods. Environ. Microbiol..

[33] Vali H, Forster O, Amarantidis G, Petersen N (1987). Magnetotactic bacteria and their magnetofossils in sediments. Earth Planet. Sci. Lett..

[34] Hanzlik M, Winklhofer M, Petersen N (1996). Spatial arrangement of chains of magnetosomes in magnetotactic bacteria. Earth Planet. Sci. Lett..

[35] Hanzlik M, Winklhofer M, Petersen N (2002). Pulsed-field-remanence measurements on individual magnetotactic bacteria. J. Magn. Magn. Mater..

[36] Eder SHK, Gigler AM, Hanzlik M, Winklhofer M (2014). Sub-micrometer-scale mapping of magnetite crystals and sulfur globules in magnetotactic bacteria using confocal Raman micro-spectrometry. PLoS ONE.

[37] Lin W, Li J, Schüler D, Jogler C, Pan Y (2009). Diversity analysis of magnetotactic bacteria in Lake Miyun, northern China, by restriction fragment length polymorphism. Syst. Appl. Microbiol..

[38] Lin W (2014). Genomic insights into the uncultured genus ‘*Candidatus* Magnetobacterium’ in the phylum *Nitrospirae*. ISME J..

[39] Li J, Liu P, Wang J, Roberts AP, Pan Y (2020). Magnetotaxis as an adaptation to enable bacterial shuttling of microbial sulfur and sulfur cycling across aquatic oxic‐anoxic interfaces. J. Geophys. Res. Biogeosci..

[40] Qian X (2019). Identification of novel species of marine magnetotactic bacteria affiliated with *Nitrospirae* phylum. Environ. Microbiol. Rep..

[41] Farina M, Lins de Barros HGP, Esquivel DMS, Danon J (1983). Ultrastructure of a magnetotactic microorganism. Biol. Cell.

[42] Winklhofer M, Abraçado LG, Davila AF, Keim CN, Lins De Barros HGP (2007). Magnetic optimization in a multicellular magnetotactic organism. Biophys. J..

[43] Keim CN (2004). Multicellular life cycle of magnetotactic prokaryotes. FEMS Microbiol. Lett..

[44] Abreu F (2007). ‘*Candidatus* Magnetoglobus multicellularis’, a multicellular, magnetotactic prokaryote from a hypersaline environment. Int. J. Syst. Evol. Microbiol.

[45] Lins U, Keim C, Evans F, Farina M, Buseck P (2007). Magnetite (Fe_3_O_4_) and greigite (Fe_3_S_4_) crystals in multicellular magnetotactic prokaryotes. Geomicrobiol. J..

[46] Wenter R, Wanner G, Schüler D, Overmann J (2009). Ultrastructure, tactic behaviour and potential for sulfate reduction of a novel multicellular magnetotactic prokaryote from North Sea sediments. Environ. Microbiol..

[47] Zhou K (2013). Adaptation of spherical multicellular magnetotactic prokaryotes to the geochemically variable habitat of an intertidal zone. Environ. Microbiol..

[48] Zhang R (2014). Characterization and phylogenetic identification of a species of spherical multicellular magnetotactic prokaryotes that produces both magnetite and greigite crystals. Res. Microbiol..

[49] Lefèvre C (2007). Characterization of Mediterranean magnetotactic bacteria. J. Ocean Univ. China.

[50] Zhou K (2012). A novel genus of multicellular magnetotactic prokaryotes from the Yellow Sea. Environ. Microbiol.

[51] Du H (2015). Temporal distributions and environmental adaptations of two types of multicellular magnetotactic prokaryote in the sediments of Lake Yuehu, China. Environ. Microbiol. Rep..

[52] Chen YR (2015). A novel species of ellipsoidal multicellular magnetotactic prokaryotes from Lake Yuehu in China. Environ. Microbiol..

[53] Chen YR (2016). Novel species and expanded distribution of ellipsoidal multicellular magnetotactic prokaryotes. Environ. Microbiol. Rep..

[54] Dong Y (2016). The detection of magnetotactic bacteria in deep sea sediments from the East Pacific Manganese Nodule Province. Environ. Microbiol. Rep..

[55] Leao P (2017). Ultrastructure of ellipsoidal magnetotactic multicellular prokaryotes depicts their complex assemblage and cellular polarity in the context of magnetotaxis. Environ. Microbiol..

[56] Teng Z (2018). Diversity and characterization of multicellular magnetotactic prokaryotes from coral reef habitats of the Paracel Islands, South China Sea. Front. Microbiol..

[57] Qian XX (2020). Juxtaposed membranes underpin cellular adhesion and display unilateral cell division of multicellular magnetotactic prokaryotes. Environ. Microbiol..

[58] Lyons NA, Kolter R (2015). On the evolution of bacterial multicellularity. Curr. Opin. Microbiol..

[59] Stolz JF, Chang SBR, Kirschvink JL (1986). Magnetotactic bacteria and single-domain magnetite in hemipelagic sediments. Nature.

[60] Petermann H, Bleil U (1993). Detection of live magnetotactic bacteria in South Atlantic deep-sea sediments. Earth Planet. Sci. Lett..

[61] Tan SM, Ismail MH, Cao B (2021). Biodiversity of magnetotactic bacteria in the tropical marine environment of Singapore revealed by metagenomic analysis. Environ. Res..

[62] Liu J (2017). Bacterial community structure and novel species of magnetotactic bacteria in sediments from a seamount in the Mariana volcanic arc. Sci. Rep..

[63] Xu C, Zhang W, Pan H, Du H, Xiao T (2018). Distribution and diversity of magnetotactic bacteria in sediments of the Yellow Sea continental shelf. J. Soils Sediment..

[64] Lefèvre CT, Frankel RB, Posfai M, Prozorov T, Bazylinski DA (2011). Isolation of obligately alkaliphilic magnetotactic bacteria from extremely alkaline environments. Environ. Microbiol..

[65] Abreu, F., Carolina, A., Araujo, V., Leão, P., Silva, K.T., and Carvalho, F.M.D. Culture-independent characterization of novel psychrophilic magnetotactic cocci from Antarctic marine sediments. *Environ Microbiol* **18**, 4426–4441 (2016). 10.1111/1462-2920.13388 27241114

[66] Nash, C. Z. *Mechanisms and Evolution of Magnetotactic Bacteria* (California Institute of Technology, 2008).

[67] Lefèvre CT (2010). Moderately thermophilic magnetotactic bacteria from hot springs in Nevada. Appl. Environ. Microbiol..

[68] Lefèvre CT (2012). Novel magnetite-producing magnetotactic bacteria belonging to the *Gammaproteobacteria*. ISME J..

[69] Liu P (2021). Identification and characterization of magnetotactic Gammaproteobacteria from a salt evaporation pool, Bohai Bay, China. Environ. Microbiol..

[70] Lyons TW, Reinhard CT, Planavsky NJ (2014). The rise of oxygen in Earth’s early ocean and atmosphere. Nature.

[71] Holland HD (1973). The oceans: a possible source of iron in iron-formations. Econ. Geol..

[72] Konhauser KO (2017). Iron formations: a global record of Neoarchaean to Palaeoproterozoic environmental history. Earth-Sci. Rev..

[73] de Baar, H. J. W. & de Jong, J. T. M. Distributions, sources and sinks of iron in seawater. in *The Biogeochemistry of Iron in Seawater* (eds. Turner, D. R. & Hunter, K. A.) 123–253 (John Wiley & Sons Ltd., 2001).

[74] Walker JCG, Brimblecombe P (1985). Iron and sulfur in the pre-biologic ocean. Precambrian Res..

[75] Habicht KS, Gade M, Thamdrup B, Berg P, Canfield DE (2002). Calibration of sulfate levels in the Archean ocean. Science.

[76] Catling DC, Zahnle KJ (2020). The Archean atmosphere. Sci. Adv..

[77] Holland HD (1985). The chemical evolution of the atmosphere and oceans. Geol. Mag..

[78] Canfield DE, Poulton SW, Narbonne GM (2007). Late-Neoproterozoic deep-ocean oxygenation and the rise of animal life. Science.

[79] Knoll AH, Nowak MA (2017). The timetable of evolution. Sci. Adv..

[80] Betts HC (2018). Integrated genomic and fossil evidence illuminates life’s early evolution and eukaryote origin. Nat. Ecol. Evol..

[81] Nutman AP, Bennett VC, Friend CRL, van Kranendonk MJ, Chivas AR (2016). Rapid emergence of life shown by discovery of 3,700-million-year-old microbial structures. Nature.

[82] Bell EA, Boehnke P, Harrison TM, Mao WL (2015). Potentially biogenic carbon preserved in a 4.1 billion-year-old zircon. Proc. Natl Acad. Sci. USA.

[83] Kopp RE, Kirschvink JL (2008). The identification and biogeochemical interpretation of fossil magnetotactic bacteria. Earth-Sci. Rev..

[84] Hounslow MW, Maher BA (1996). Quantitative extraction and analysis of carriers of magnetization in sediments. Geophys. J. Int..

[85] Montgomery P, Hailwood EA, Gale AS, Burnett JA (1998). The magnetostratigraphy of Coniacian late Campanian chalk sequences in southern England. Earth Planet. Sci. Lett..

[86] Blankenship RE (2010). Early evolution of photosynthesis. Plant Physiol..

[87] Williams BP, Aubry S, Hibberd JM (2012). Molecular evolution of genes recruited into C 4 photosynthesis. Trends Plant Sci..

[88] Ilbert M, Bonnefoy V (2013). Insight into the evolution of the iron oxidation pathways. Biochim. Biophys. Acta Bioenerg..

[89] Camacho A, Walter XA, Picazo A, Zopfi J (2017). Photoferrotrophy: remains of an ancient photosynthesis in modern environments. Front. Microbiol..

[90] Simmons, S. L. & Edwards, K. J. Geobiology of magnetotactic bacteria. In *Magnetoreception and Magnetosomes in Bacteria* (ed. Schüler, D.) 77–102 (Springer, 2006).

[91] Strbak O, Dobrota D (2019). Archean iron-based metabolism analysis and the photoferrotrophy-driven hypothesis of microbial magnetotaxis origin. Geomicrobiol. J..

[92] Muñoz D (2020). Magnetosomes could be protective shields against metal stress in magnetotactic bacteria. Sci. Rep..

[93] Monteil CL (2018). Genomic study of a novel magnetotactic *Alphaproteobacteria* uncovers the multiple ancestry of magnetotaxis. Environ. Microbiol..

[94] Du H (2019). Magnetosome gene duplication as an important driver in the evolution of magnetotaxis in the *Alphaproteobacteria*. mSystems.

[95] Monteil CL (2020). Repeated horizontal gene transfers triggered parallel evolution of magnetotaxis in two evolutionary divergent lineages of magnetotactic bacteria. ISME J..

[96] Natan, E., Fitak, R. R., Werber, Y. & Vortman, Y. Symbiotic magnetic sensing: raising evidence and beyond. *Philos. Trans. R. Soc. Lond. B* **375**, 20190595 (2020). 10.1098/rstb.2019.0595 PMC7435164 32772668

[97] Natan E, Vortman Y (2017). The symbiotic magnetic-sensing hypothesis: do magnetotactic bacteria underlie the magnetic sensing capability of animals?. Mov. Ecol..

[98] Simon RA (2021). Magnetotactic bacteria from the human gut microbiome associated with orientation and navigation regions of the brain. J. Oceanol. Limnol..

[99] Bellinger, M. R. et al. Conservation of magnetite biomineralization genes in all domains of life and implications for magnetic sensing. *Proc. Natl. Acad. Sci. USA* **119**, e2108655119 (2022). 10.1073/pnas.2108655119 PMC8784154 35012979

[100] Yuan W, Zhou H, Yang Z, Hein JR, Yang Q (2020). Magnetite magnetofossils record biogeochemical remanent magnetization in hydrogenetic ferromanganese crusts. Geology.

[101] Jiang XD (2020). Characterization and quantification of magnetofossils within abyssal manganese nodules from the Western Pacific Ocean and implications for nodule formation. Geochem. Geophys. Geosyst..

[102] Hassan Mbin (2020). Presence of biogenic magnetite in ferromanganese nodules. Environ. Microbiol. Rep..

[103] Oda H, Nakasato Y, Usui A (2018). Characterization of marine ferromanganese crust from the Pacific using residues of selective chemical leaching: Identification of fossil magnetotactic bacteria with FE-SEM and rock magnetic methods. Earth, Planets Space.

[104] Kirschvink JL, Chang SBR (1984). Ultrafine-grained magnetite in deep-sea sediments—possible bacterial magnetofossils. Geology.

[105] Petersen N, von Dobeneck T, Vali H (1986). Fossil bacterial magnetite in deep-sea sediments from the South-Atlantic Ocean. Nature.

[106] Li J (2010). Biomineralization, crystallography and magnetic properties of bullet-shaped magnetite magnetosomes in giant rod magnetotactic bacteria. Earth Planet. Sci. Lett..

[107] Moskowitz BM, Frankel RB, Bazylinski DA (1993). Rock magnetic criteria for the detection of biogenic magnetite. Earth Planet. Sci. Lett..

[108] Chang L (2013). Low-temperature magnetic properties of pelagic carbonates: oxidation of biogenic magnetite and identification of magnetosome chains. J. Geophys. Res. Solid Earth.

[109] Chang L, Heslop D, Roberts AP, Rey D, Mohamed KJ (2016). Discrimination of biogenic and detrital magnetite through a double Verwey transition temperature. J. Geophys. Res. Solid Earth.

[110] Jackson MJ, Moskowitz B (2021). On the distribution of Verwey transition temperatures in natural magnetites. Geophys. J. Int.

[111] Pike CR, Roberts AP, Verosub KL (1999). Characterizing interactions in fine magnetic particle systems using first order reversal curves. J. Appl. Phys..

[112] Roberts AP, Pike CR, Verosub KL (2000). First-order reversal curve diagrams: a new tool for characterizing the magnetic properties of natural samples. J. Geophys. Res. Solid Earth.

[113] Roberts AP, Heslop D, Zhao X, Pike CR (2014). Understanding fine magnetic particle systems through use of first-order reversal curve diagrams. Rev. Geophys..

[114] Dunin-Borkowski RE (1998). Magnetic microstructure of magnetotactic bacteria by electron holography. Science.

[115] Pan YX (2005). Rock magnetic properties of uncultured magnetotactic bacteria. Earth Planet. Sci. Lett..

[116] Roberts AP (2011). Magnetotactic bacterial abundance in pelagic marine environments is limited by organic carbon flux and availability of dissolved iron. Earth Planet. Sci. Lett..

[117] Chen AP, Egli R, Moskowitz BM (2007). First-order reversal curve (FORC) diagrams of natural and cultured biogenic magnetic particles. J. Geophys. Res. Solid Earth.

[118] Egli R, Chen AP, Winklhofer M, Kodama KP, Horng C-S (2010). Detection of noninteracting single domain particles using first-order reversal curve diagrams. Geochem. Geophys. Geosyst..

[119] Li J, Wu W, Liu Q, Pan Y (2012). Magnetic anisotropy, magnetostatic interactions and identification of magnetofossils. Geochem. Geophys. Geosyst..

[120] Amor M (2022). Key signatures of magnetofossils elucidated by mutant magnetotactic bacteria and micromagnetic calculations. J. Geophys. Res. Solid Earth.

[121] Charilaou M, Winklhofer M, Gehring AU (2011). Simulation of ferromagnetic resonance spectra of linear chains of magnetite nanocrystals. J. Appl. Phys..

[122] Weiss BP (2004). Ferromagnetic resonance and low-temperature magnetic tests for biogenic magnetite. Earth Planet. Sci. Lett..

[123] Kopp RE (2006). Ferromagnetic resonance spectroscopy for assessment of magnetic anisotropy and magnetostatic interactions: a case study of mutant magnetotactic bacteria. J. Geophys. Res. Solid Earth.

[124] Kodama KP, Moeller RE, Bazylinski DA, Kopp RE, Chen AP (2013). The mineral magnetic record of magnetofossils in recent lake sediments of Lake Ely, PA. Glob. Planet. Change.

[125] Chang L (2014). Magnetic detection and characterization of biogenic magnetic minerals: a comparison of ferromagnetic resonance and first‐order reversal curve diagrams. J. Geophys. Res. Solid Earth.

[126] Kind J, Gehring AU, Winklhofer M, Hirt AM (2011). Combined use of magnetometry and spectroscopy for identifying magnetofossils in sediments. Geochem. Geophys. Geosyst..

[127] Blattmann TM (2020). Ferromagnetic resonance of magnetite biominerals traces redox changes. Earth Planet. Sci. Lett..

[128] Larrasoaña JC (2012). Magnetotactic bacterial response to Antarctic dust supply during the Palaeocene-Eocene thermal maximum. Earth Planet. Sci. Lett..

[129] Muxworthy AR, Williams W (2006). Critical single-domain/multidomain grain sizes in noninteracting and interacting elongated magnetite particles: implications for magnetosomes. J. Geophys. Res. Solid Earth.

[130] Chang L (2018). Coupled microbial bloom and oxygenation decline recorded by magnetofossils during the Palaeocene–Eocene thermal maximum. Nat. Commun..

[131] Berndt TA, Chang L, Pei Z (2020). Mind the gap: towards a biogenic magnetite palaeoenvironmental proxy through an extensive finite-element micromagnetic simulation. Earth Planet. Sci. Lett..

[132] Heslop D (2013). Quantifying magnetite magnetofossil contributions to sedimentary magnetizations. Earth Planet. Sci. Lett..

[133] Kruiver PP, Dekkers MJ, Heslop D (2001). Quantification of magnetic coercivity components by the analysis of acquisition curves of isothermal remanent magnetisation. Earth Planet. Sci. Lett..

[134] Egli R (2003). Analysis of the field dependence of remanent magnetization curves. J. Geophys. Res. Solid Earth.

[135] Zhao X, Fujii M, Suganuma Y, Zhao X, Jiang Z (2018). Applying the Burr type XII distribution to decompose remanent magnetization curves. J. Geophys. Res. Solid Earth.

[136] Egli R (2004). Characterization of individual rock magnetic components by analysis of remanence curves. 2. Fundamental properties of coercivity distributions. Phys. Chem. Earth.

[137] Heslop D, Dillon M (2007). Unmixing magnetic remanence curves without a priori knowledge. Geophys. J. Int.

[138] Usui Y, Yamazaki T, Saitoh M (2017). Changing abundance of magnetofossil morphologies in pelagic red clay around Minamitorishima, Western North Pacific. Geochem. Geophys. Geosyst..

[139] Lascu I (2015). Magnetic unmixing of first-order reversal curve diagrams using principal component analysis. Geochem. Geophys. Geosyst..

[140] Harrison RJ (2018). An improved algorithm for unmixing first‐order reversal curve diagrams using principal component analysis. Geochem. Geophys. Geosyst..

[141] Channell JET (2016). Magnetic record of deglaciation using FORC-PCA, sortable-silt grain size, and magnetic excursion at 26 ka, from the Rockall Trough (NE Atlantic). Geochem. Geophys. Geosyst..

[142] Roberts AP (2018). Signatures of reductive magnetic mineral diagenesis from unmixing of first-order reversal curves. J. Geophys. Res. Solid Earth.

[143] Yamazaki T, Fu W, Shimono T, Usui Y (2020). Unmixing biogenic and terrigenous magnetic mineral components in red clay of the Pacific Ocean using principal component analyses of first-order reversal curve diagrams and paleoenvironmental implications. Earth, Planets Space.

[144] Egli R (2004). Characterization of individual rock magnetic components by analysis of remanence curves, 1. Unmixing natural sediments. Stud. Geophys. Geod..

[145] Qian Y (2020). Assessment and integration of bulk and component-specific methods for identifying mineral magnetic assemblages in environmental magnetism. J. Geophys. Res. Solid Earth.

[146] Inoue K, Yamazaki T, Usui Y (2021). Influence of magnetofossils on paleointensity estimations inferred from principal component analyses of first‐order reversal curve diagrams for sediments from the Western Equatorial Pacific. Geochem. Geophys. Geosyst..

[147] Wagner CL (2021). Diversification of iron-biomineralizing organisms during the Paleocene-Eocene thermal maximum: evidence from quantitative unmixing of magnetic signatures of conventional and giant magnetofossils. Paleoceanogr. Paleoclimatol.

[148] Zhang Q (2021). Magnetotactic bacterial activity in the North Pacific Ocean and its relationship to Asian dust inputs and primary productivity since 8.0 Ma. Geophys. Res. Lett..

[149] Hesse PP (1994). Evidence for bacterial palaeoecological origin of mineral magnetic cycles in oxic and sub-oxic Tasman Sea sediments. Mar. Geol..

[150] Yamazaki T, Kawahata H (1998). Organic carbon flux controls the morphology of magnetofossils in marine sediments. Geology.

[151] He K, Pan Y (2020). Magnetofossil abundance and diversity as paleoenvironmental proxies: a case study from Southwest Iberian margin sediments. Geophys. Res. Lett..

[152] Lean CMB, McCave IN (1998). Glacial to interglacial mineral magnetic and palaeoceanographic changes at Chatham Rise, SW Pacific Ocean. Earth Planet. Sci. Lett..

[153] Yamazaki T (2020). Reductive dissolution of biogenic magnetite. Earth, Planets Space.

[154] Mathias GL (2021). A multi-proxy approach to unravel late Pleistocene sediment flux and bottom water conditions in the western South Atlantic Ocean. Paleoceanogr. Paleoclimatol.

[155] Rodelli D (2019). Diagenetic fate of biogenic soft and hard magnetite in chemically stratified sedimentary environments of Mamangua Ria, Brazil. J. Geophys. Res. Solid Earth.

[156] Ouyang T (2014). Variable remanence acquisition efficiency in sediments containing biogenic and detrital magnetites: implications for relative paleointensity signal recording. Geochem. Geophys. Geosyst..

[157] Chen L (2017). Remanence acquisition efficiency in biogenic and detrital magnetite and recording of geomagnetic paleointensity. Geochem. Geophys. Geosyst..

[158] Li J (2021). Paleomagnetism of a sediment core taken from the Ontong-Java Plateau: for better understanding of the role of biogenic magnetite in geomagnetic paleointensity recording. Preprint.

[159] Tarduno JA, Tian WL, Wilkison S (1998). Biogeochemical remanent magnetization in pelagic sediments of the western equatorial Pacific Ocean. Geophys. Res. Lett..

[160] Yamazaki T, Suzuki Y, Kouduka M, Kawamura N (2019). Dependence of bacterial magnetosome morphology on chemical conditions in deep-sea sediments. Earth Planet. Sci. Lett..

[161] Vasiliev I (2008). Putative greigite magnetofossils from the Pliocene epoch. Nat. Geosci..

[162] Pósfai M (2001). Crystal-size distributions and possible biogenic origin of Fe sulfides. Eur. J. Mineral..

[163] Chang L (2014). Identification and environmental interpretation of diagenetic and biogenic greigite in sediments: a lesson from the Messinian Black Sea. Geochem. Geophys. Geosyst..

[164] Bazylinski DA, Blakemore RP (1983). Nitrogen fixation (acetylene reduction) in *Aquaspirillum magnetotacticum*. Curr. Microbiol..

[165] Jiang W (2002). Submerged culture of *Magnetospirillum gryphiswaldense* under N_2_-fixing condition and regulation of activity of nitrogen fixation. Chin. Sci. Bull..

[166] Bazylinski DA (2004). Chemolithoautotrophy in the marine, magnetotactic bacterial strains MV-1 and MV-2. Arch. Microbiol..

[167] Schultheiss D, Handrick R, Jendrossek D, Hanzlik M, Schüler D (2005). The presumptive magnetosome protein Mms16 is a poly(3-hydroxybutyrate) granule-bound protein (phasin) in *Magnetospirillum gryphiswaldense*. J. Bacteriol..

[168] Rivas-Lamelo S (2017). Magnetotactic bacteria as a new model for P sequestration in the ferruginous Lake Pavin. Geochem. Perspect. Lett..

[169] Schulz-Vogt HN (2019). Effect of large magnetotactic bacteria with polyphosphate inclusions on the phosphate profile of the suboxic zone in the Black Sea. ISME J..

[170] Tanaka M (2016). Biomagnetic recovery and bioaccumulation of selenium granules in magnetotactic bacteria. Appl. Environ. Microbiol..

[171] Isambert A, Menguy N, Larquet E, Guyot F, Valet J-P (2007). Transmission electron microscopy study of magnetites in a freshwater population of magnetotactic bacteria. Am. Mineral..

[172] Cornejo E, Subramanian P, Li Z, Jensen GJ, Komeili A (2016). Dynamic remodeling of the magnetosome membrane is triggered by the initiation of biomineralization. MBio.

[173] Li J (2020). Bullet-shaped magnetite biomineralization within a magnetotactic Deltaproteobacterium: implications for magnetofossil identification. J. Geophys. Res. Biogeosci..

[174] Bazylinski DA (1995). Structure and function of the bacterial magnetosome. ASM N..

[175] Simmons SL, Bazylinski DA, Edwards KJ (2006). South-seeking magnetotactic bacteria in the Northern Hemisphere. Science.

[176] Faivre D, Fischer A, Garcia-Rubio I, Mastrogiacomo G, Gehring AU (2010). Development of cellular magnetic dipoles in magnetotactic bacteria. Biophys. J..

[177] Coale KH (2004). Southern ocean iron enrichment experiment: carbon cycling in high- and low-Si waters. Science.

[178] Jacq V, Ridame C, L’Helguen S, Kaczmar F, Saliot A (2014). Response of the unicellular diazotrophic cyanobacterium *Crocosphaera watsonii* to iron limitation. PLoS ONE.

[179] Martínez-García A, Winckler G (2014). Iron fertilization in the glacial ocean. PAGES Mag..

[180] Street JH, Paytan A (2005). Iron, phytoplankton growth, and the carbon cycle. Met. Ions Biol. Syst..

[181] Aumont O, Bopp L (2006). Globalizing results from ocean in situ iron fertilization studies. Glob. Biogeochem. Cycles.

[182] Wambeke Fvan (2008). Nutrient limitation of primary productivity in the Southeast Pacific. Biogeosciences.

[183] Schoffman H, Lis H, Shaked Y, Keren N (2016). Iron-nutrient interactions within phytoplankton. Front. Plant Sci..

[184] Amor M, Tharaud M, Gélabert A, Komeili A (2020). Single-cell determination of iron content in magnetotactic bacteria: implications for the iron biogeochemical cycle. Environ. Microbiol..

[185] Vainshtein M, Suzina N, Sorokin V (1997). A new type of magnet-sensitive inclusions in cells of photosynthetic purple bacteria. Syst. Appl. Microbiol..

[186] Sakaguchi T, Arakaki A, Matsunaga T (2002). *Desulfovibrio magneticus* sp. nov., a novel sulfate-reducing bacterium that produces intracellular single-domain-sized magnetite particles. Int. J. Syst. Evol. Microbiol..

[187] Rahn-Lee L (2015). A genetic strategy for probing the functional diversity of magnetosome formation. PLoS Genet..

[188] Grant, C. R. et al. Distinct gene clusters drive formation of ferrosome organelles in bacteria. *Nature* (2022) 10.1038/s41586-022-04741-x. 10.1038/s41586-022-04741-x PMC10906721 35585231

[189] Amor M (2020). Magnetotactic bacteria accumulate a large pool of iron distinct from their magnetite crystals. Appl. Environ. Microbiol..

[190] Lefèvre CT, Frankel RB, Abreu F, Lins U, Bazylinski DA (2011). Culture‐independent characterization of a novel, uncultivated magnetotactic member of the *Nitrospirae* phylum. Environ. Microbiol..

[191] Lin W, Li J, Pan Y (2012). Newly isolated but uncultivated magnetotactic bacterium of the phylum *Nitrospirae* from Beijing, China. Appl. Environ. Microbiol..

[192] Spring S, Bazylinski DA (2006). Magnetotactic bacteria. Prokaryotes.

[193] Kolinko S, Richter M, Gloeckner F-O, Brachmann A, Schüler D (2016). Single-cell genomics of uncultivated deep-branching magnetotactic bacteria reveals a conserved set of magnetosome genes. Environ. Microbiol..

[194] Sen J (1929). The role of associated nitrogen-fixing bacteria on nitrogen nutrition of cereal crops. Agric. J. India.

[195] Rubin-Blum M, Dubilier N, Kleiner M (2019). Genetic evidence for two carbon fixation pathways (the Calvin-Benson-Bassham Cycle and the Reverse Tricarboxylic Acid Cycle) in symbiotic and free-living bacteria. Msphere.

[196] Hoppenrath, M., Elbrächter, M. & Drebes, G. *Marine Phytoplankton: Selected Microphytoplankton Species from the North Sea Around Helgoland and Sylt* (E. Schweizerbart’sche Verlagsbuchhandlung, 2009).

[197] Matin A (1978). Organic nutrition of chemolithotrophic bacteria. Annu. Rev. Microbiol..

[198] Paoli GC, Morgan NS, Tabita FR, Shively JM (1995). Expression of the cbbLcbbS and cbbM genes and distinct organization of the cbb Calvin cycle structural genes of *Rhodobacter capsulatus*. Arch. Microbiol..

[199] Williams TJ, Zhang CL, Scott JH, Bazylinski DA (2006). Evidence for autotrophy via the reverse tricarboxylic acid cycle in the marine magnetotactic coccus strain MC-1. Appl. Environ. Microbiol..

[200] Zhang W, Wang Y, Liu L, Pan Y, Lin W (2021). Identification and genomic characterization of two previously unknown magnetotactic *Nitrospirae*. Front. Microbiol..

[201] Monteil CL (2021). Intracellular amorphous Ca-carbonate and magnetite biomineralization by a magnetotactic bacterium affiliated to the Alphaproteobacteria. ISME J..

[202] Arakaki A, Takeyama H, Tanaka T, Matsunaga T (2002). Cadmium recovery by a sulfate-reducing magnetotactic bacterium, *Desulfovibrio magneticus* RS-1, using magnetic separation. Appl. Biochem. Biotech..

[203] Tajer-Mohammad-Ghazvini P (2016). Cobalt separation by *Alphaproteobacterium* MTB-KTN90: Magnetotactic bacteria in bioremediation. Bioprocess Biosyst. Eng..

[204] Tanaka M, Arakaki A, Staniland SS, Matsunaga T (2010). Simultaneously discrete biomineralization of magnetite and tellurium nanocrystals in magnetotactic bacteria. Appl. Environ. Microbiol..

[205] Sannigrahi S, Suthindhiran K (2019). Metal recovery from printed circuit boards by magnetotactic bacteria. Hydrometallurgy.

[206] Postec A (2012). Magnetotactic bacteria in microcosms originating from the French Mediterranean coast subjected to oil industry activities. Microb. Ecol..

[207] Sewell RBS, Fage L (1948). Minimum oxygen layer in the ocean. Nature.

[208] Cavicchioli R (2019). Scientists’ warning to humanity: microorganisms and climate change. Nat. Rev. Microbiol..

[209] Ulloa O, Canfield DE, DeLong EF, Letelier RM, Stewart FJ (2012). Microbial oceanography of anoxic oxygen minimum zones. Proc. Natl Acad. Sci. USA.

[210] Schmidtko S, Stramma L, Visbeck M (2017). Decline in global oceanic oxygen content during the past five decades. Nature.

[211] Breitburg, D. et al. Declining oxygen in the global ocean and coastal waters. *Science* **359**, eaam7240 (2018). 10.1126/science.aam7240 29301986

[212] Bertagnolli AD, Stewart FJ (2018). Microbial niches in marine oxygen minimum zones. Nat. Rev. Microbiol..

[213] Wright JJ, Konwar KM, Hallam SJ (2012). Microbial ecology of expanding oxygen minimum zones. Nat. Rev. Microbiol..

[214] Paulmier A, Ruiz-Pino D (2009). Oxygen minimum zones (OMZs) in the modern ocean. Progr. Oceanogr..

[215] Ulloa O, Pantoja S (2009). The oxygen minimum zone of the eastern South Pacific. Deep-Sea Res. II.

[216] Stevens H, Ulloa O (2008). Bacterial diversity in the oxygen minimum zone of the eastern tropical South Pacific. Environ. Microbiol..

[217] Lavik G (2009). Detoxification of sulphidic African shelf waters by blooming chemolithotrophs. Nature.

[218] Walsh DA (2009). Metagenome of a versatile chemolithoautotroph from expanding oceanic dead zones. Science.

[219] Canfield DE (2010). A cryptic sulfur cycle in oxygen-minimum-zone waters off the Chilean coast. Science.

[220] Rhoads DC, Mulsow SG, Gutschick R, Baldwin CT, Stolz JF (1991). The dysaerobic zone revisited: a magnetic facies?. Geol. Soc. Lond. Spec. Publ..

[221] Brune A, Frenzel P, Cypionka H (2000). Life at the oxic-anoxic interface: microbial activities and adaptations. FEMS Microbiol. Rev..

[222] Monteil CL (2019). Ectosymbiotic bacteria at the origin of magnetoreception in a marine protist. Nat. Microbiol..

[223] Reitner J (2005). Concretionary methane-seep carbonates and associated microbial communities in Black Sea sediments. Palaeogeogr. Palaeoclimatol. Palaeoecol..

[224] Dufour SC (2014). Magnetosome-containing bacteria living as symbionts of bivalves. ISME J..

